# Profiling target engagement and cellular uptake of cRGD-decorated clinical-stage core-crosslinked polymeric micelles

**DOI:** 10.1007/s13346-022-01204-8

**Published:** 2022-07-11

**Authors:** Federica De Lorenzi, Larissa Yokota Rizzo, Rasika Daware, Alessandro Motta, Maike Baues, Matthias Bartneck, Michael Vogt, Marc van Zandvoort, Leonard Kaps, Qizhi Hu, Marielle Thewissen, Luca Casettari, Cristianne J. F. Rijcken, Fabian Kiessling, Alexandros Marios Sofias, Twan Lammers

**Affiliations:** 1 Department of Nanomedicine and Theranostics, Institute for Experimental Molecular Imaging (ExMI), RWTH Aachen University Clinic, Aachen, Germany; 2 Clinic for Gastroenterology, Metabolic Diseases and Internal Intensive Care Medicine (Internal Medicine III), RWTH Aachen University Clinic, Aachen, Germany; 3 Interdisciplinary Center for Clinical Research (IZKF), RWTH Aachen University Clinic, Aachen, Germany; 4 Institute for Molecular Cardiovascular Research (IMCAR), RWTH Aachen University Clinic, Aachen, Germany; 5 Department of Genetics and Cell Biology, School for Cardiovascular Diseases (CARIM), School for Oncology and Reproduction (GROW), School for Mental Health and Neuroscience (MHeNS), Department of Genetics and Cell Biology, Maastricht University, Maastricht, The Netherlands; 6 Department of Medicine, University Medical Center Mainz, Mainz, Germany; 7 Institute of Translational Immunology and Research Center for Immune Therapy, University Medical Center Mainz, Mainz, Germany; 8 Cristal Therapeutics, Maastricht, The Netherlands; 9 Department of Biomolecular Sciences, University of Urbino Carlo Bo, Urbino, Italy; 10 Institute for Experimental Molecular Imaging (ExMI), RWTH Aachen University Clinic, Aachen, Germany; 11 Fraunhofer Institute for Digital Medicine MEVIS, Bremen, Germany; 12 Mildred Scheel School of Oncology (MSSO), Center for Integrated Oncology, University Hospital Aachen, Aachen, Germany; 13 Department of Circulation and Medical Imaging, Faculty of Medicine and Health Sciences, Norwegian University of Science and Technology (NTNU), Trondheim, Norway

**Keywords:** Nanomedicine, Tumor targeting, Nanoparticles, Core-crosslinked polymeric micelles, Cyclic arginine-glycine-aspartic acid (cRGD), CriPec^®^

## Abstract

Polymeric micelles are increasingly explored for tumor-targeted drug delivery. CriPec® technology enables the generation of core‐crosslinked polymeric micelles (CCPMs) based on thermosensitive (mPEG-b-pHPMAmLac_n_) block copolymers, with high drug loading capacity, tailorable size, and controlled drug release kinetics. In this study, we decorated clinical-stage CCPM with the α_v_β_3_ integrin-targeted cyclic arginine-glycine-aspartic acid (cRGD) peptide, which is one of the most well-known active targeting ligands evaluated preclinically and clinically. Using a panel of cell lines with different expression levels of the α_v_β_3_ integrin receptor and exploring both static and dynamic incubation conditions, we studied the benefit of decorating CCPM with different densities of cRGD. We show that incubation time and temperature, as well as the expression levels of α_v_β_3_ integrin by target cells, positively influence cRGD-CCPM uptake, as demonstated by immunofluorescence staining and fluorescence microscopy. We demonstrate that even very low decoration densities (i.e., 1 mol % cRGD) result in increased engagement and uptake by target cells as compared to peptide-free control CCPM, and that high cRGD decoration densities do not result in a proportional increase in internalization. In this context, it should be kept in mind that a more extensive presence of targeting ligands on the surface of nanomedicines may affect their pharmacokinetic and biodistribution profile. Thus, we suggest a relatively low cRGD decoration density as most suitable for in vivo application.

**Graphical Abstract:**

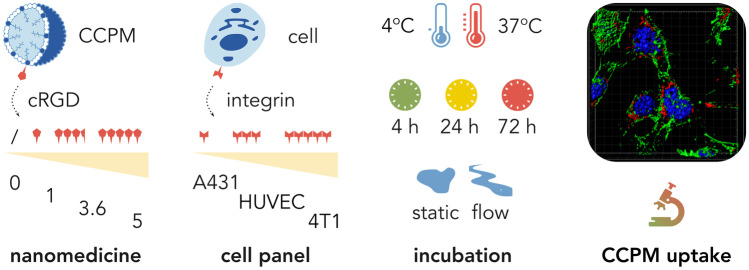

**Supplementary information:**

The online version contains supplementary material available at 10.1007/s13346-022-01204-8.

## Introduction

Polymeric micelles are versatile nanocarriers, extensively used for the encapsulation and delivery of hydrophobic drugs. Such drugs can be entrapped and stabilized either via physical (e.g., hydrophobic interactions and π-π stacking) or chemical interactions (e.g., covalent core-crosslinking) [1, 2]. Formulations based on CriPec^®^ technology are core-crosslinked polymeric micelles (CCPMs) based on thermosensitive methoxy poly(ethylene glycol)-*b*-poly[N-(2-hydroxypropyl) methacrylamide lactate] (mPEG-b-pHPMAmLac_n_) block copolymers. During the manufacturing of CriPec^®^ CCPM, the block copolymers self-assemble into core–shell structures in aqueous solutions and are then crosslinked in the micellar core by means of free radical polymerization. During this process, hydrophobic drug molecules are co-crosslinked, resulting in covalent attached in the stabilized micellar core via hydrolysable linkages that enable release of native drug molecules at the target site with predetermined kinetics [3].

Covalent core-crosslinking results in enhanced in vivo stability and prolonged circulation half-life times, allowing for an efficient accumulation at the target site [1, 4, 5], in contrast to conventional polymeric micelles that typically disintegrate faster in systemic circulation upon intravenous (i.v.) administration. These benefits have been apparent in preclinical image-guided drug delivery set-ups where fluorescence-labeled CCPMs were tracked in vivo via hybrid fluorescence tomography – computed tomography. In this set-up, CCPM achieved a tumor accumulation of 18.6%ID/g at 48 h post i.v. injection in a triple-negative breast cancer model in mice [6]. Extending preclinical evaluation, CriPec®-based CCPM technology has been clinically trialed. Docetaxel-entrapped CCPM, also known as CPC634, is the clinically most advanced nanomedicine manufactured by Cristal Therapeutics. CPC634 has undergone phases I and II clinical trials in the Netherlands, Belgium, and UK, for assessing the micelles’ pharmacokinetic (PK), biodistribution (BD), and accumulation in solid tumors and metastases (NCT03712423, NCT02442531), as well as to evaluate its safety and efficacy in platinum-resistant ovarian cancer (NCT03742713) [7–9]. Of note, the administration of CPC634 resulted in a four-fold higher tumor accumulation of docetaxel in advanced solid tumors in patients, as compared to the administration of free docetaxel [7].

All above-mentioned preclinical and clinical studies have been executed employing passively targeted CCPM. Passive targeting to malignant lesions has been described to occur due to disease-specific hyperpermeable blood vessels and defective lymphatic drainage. The combination of passive barrier-crossing and retention has been described by Matsumura and Maeda in 1986 as the enhanced permeability and retention (EPR) effect [10]. While in the last couple of decades passive targeting has been mostly attributed to vascular leakiness, novel image-guided research has discovered the extravasation of passive targeting nanomedicines to be a cumulative effect of vascular bursts, myeloid cell-dependent vascular ruptures, and endothelial transcytosis [11–14]. Furthermore, the retention of nanomedicines at malignant sites has been described to be secondary to phagocytic uptake by tumor-associated macrophages (TAMs) as a mechanism of nanoparticle retention [15].

In addition to EPR and other passive targeting mechanisms, decoration of nanomedicines with targeting ligands offers the possibility for active targeting by capitalizing on the molecular specificity of a ligand or an antibody/antibody fragment for a receptor upregulated by the cell population target. Such nanoparticle surface decorations can potentiate targeting of a drug to a specific tissue, cell type, or subcellular compartment [16]. Even though this concept appears straightforwardly beneficial, actively targeted nanomedicines have not yet been approved for use in the clinic, due to overall suboptimal in vivo performance and unfavorable PK profiles, due to poor biological barriers penetration, protein corona formation, and recognition by the mononuclear phagocyte system (MPS) [16, 17]. In this regard, various parameters during the preparation procedure may crucially affect the in vivo performance of active targeting nanomedicine, e.g., ligand decoration procedures, surface decoration density, ligand decoration density, and exposure of the ligand at the outer shell of the nanoparticle [18–21].

In this study, we decorated CCPM with an arginine-glycine-aspartic acid peptide (i.e., RGD; note that the cyclic RGDfK pentapeptide was used), as it is one of the most well-known active targeting ligands tested both preclinically and clinically [22–28] and holds promise for targeting not only cancer cells but also activated endothelial cells [29], stromal cells [30], and immune cells [31]. In addition, cRGD has been shown to increase the accumulation of nanomedicines in tumors by promoting transcytosis [32, 33]. By producing CCPM with three different decoration densities of cRGD (0, 1, 3.6, and 5 mol%) and a control composition without cRGD decoration, we aimed at assessing the biological activity of the ligand when anchored on the nanoformulation, as well as at examining whether higher decoration densities are meaningful for improving target binding and internalization. Of note, too high levels of ligand decoration density are known to deteriorate in vivo performance due to high recognition by phagocytes and off-target deposition in clearance organs [34, 35].

Our experimental set-up included the incubation of four cell lines/primary cells (Α431, HUVEC, activated HUVEC, 4T1) that are well-known to display different α_v_β_3_ integrin expression levels (Fig. S1 – α_v_β_3_ integrin receptor-target of cRGD ligand) with all four CCPM variations (0, 1, 3.6, and 5 mol% cRGD), at various temperatures (4 and 37 °C), time points (4, 24, and 72 h), and incubation conditions (static and under flow). The assessment of CCPM engagement, target binding, and uptake by cells was validated via histology, fluorescence microscopy, multiphoton microscopy, and flow cytometry. The outcomes of this study improve our understanding on the effect of ligand decoration densities in targeting a given biological system, and they promote progress in developing actively targeted cancer nanomedicines.

## Materials and methods

### Rhodamine-labeled core-crosslinked polymeric micelles

Rhodamine-labeled block copolymer was synthesized by conjugating rhodamine B (excitation = 550 nm) to the terminal hydroxyl groups of polymer lactate side chains, via formation of an ester bond. Partially methacrylated mPEG_5000_-b-pHPMAmDP_1_DP_2_ block copolymer (21.5 kDa, 10 mol% methacrylated, 0.140 mmol) was reacted with rhodamine B (0.698 mmol) using N,N′-dicyclohexylcarbodiimide (DCC) (0.698 mmol) and 4-(dimethylamino)pyridine (DMAP) (0.698 mmol) in dichloromethane (DCM) (30 mL) at room temperature. After 24 h, DCM was evaporated and the remaining reaction mixture was dissolved in milliQ water (150 mL), followed by dialysis (MWCO 12–14 kDa) against acetonitrile (ACN)/milliQ water (50v/50v) at 4 °C to remove unreacted rhodamine molecules, and subsequently freeze-dried to obtain rhodamine-labeled block copolymer as a pink powder. Consequently, a mixture of synthesized rhodamine-labeled block copolymer (23 w%) and methacrylated mPEG_5000_-b-pHPMAmDP_1_DP_2_ block copolymer (77 w%) was used to synthesize rhodamine-labeled core-crosslinked polymeric micelles, following a previously reported protocol [1]. DCC and DMAP were purchased from Sigma-Aldrich. Dichloromethane DCM and ACN were obtained from Actuall Chemicals. Azide-PEG_5000_-OH was purchased from Rapp Polymere GmbH. Rhodamine B was purchased from Acros Organics. To functionalize CPPM with cRGD, the cRGDfk-targeting ligand was conjugated to the azide moiety of the nanoparticles via BCN conjugation [1, 4, 36]. Four batches of cRGD-conjugated rhodamine-labeled CriPec^®^ (empty) core-crosslinked polymeric micelles (CCPM) were provided by Cristal Therapeutics (Maastricht, The Netherlands), containing 0, 1, 3.6, and 5 mol% cRGD. All micelles were characterized via DLS, displaying a small size (35–40 nm in diameter) and a narrow size distribution (polydispersity index between 0.15 and 0.34). The polymer content (expressed as polymer concentration; mg/ml) of each nanoformulation was evaluated via lactic acid detection and a conversion rate of 3.4. CCPMs were dispersed in 180 mM HEPES buffer pH 7.0, hence, batches were stored at 4 °C.

#### In vitro cell culture

A431 epidermoid squamous cancer cells were purchased from ATTC^®^ (Manassas, VA) and cultured in RPMI medium (Invitrogen; Darmstadt, Germany) supplemented with 10% FCS (Fetal Calf Serum; Invitrogen, Darmstadt, Germany) and 1% penicilinin/streptavidin (Pen/Strept; Invitrogen, Darmstadt, Germany). HUVECs (human umbilical vein endothelial cells) were purchased from Promocell^®^ (Heidelberg, Germany) and cultured with endothelial cell growth medium (Endopan 3 Pan-Biotech^®^ 500 ml) supplemented with 15 ml FCS, 0.1 ml hydrocortison, 0.5 ml EGF, 0.5 ml ascorbic acid, 0.5 ml VEGF, 0.5 ml FGF2, 0.5 ml heparin, and 5 ml P/S, 5 ml gentamicin sulfate). As primary cells, HUVECs were only used up to passage number 8. 4T1 murine triple-negative breast cancer cells were purchased from ATTC^®^ (Manassas, VA) and cultured in RPMI medium (Invitrogen; Darmstadt, Germany) supplemented with 10% FCS (Fetal Calf Serum; Invitrogen, Darmstadt, Germany) and 1% penicillin/streptavidin (Pen/Strept; Invitrogen, Darmstadt, Germany). Cell passaging was performed following standard cell culture protocols in T75 cell culture flasks (Cell Star, Greiner, Germany) upon 70–80% confluency. Cultures were maintained in a 37 °C incubator with 5% CO_2_ and 95% relative humidity.

#### Nanoparticle uptake assay under static cultivation conditions

The cell uptake of CCPM was tested at different incubation times (4, 24, and 72 h) and at different incubation temperature (4 °C, 37 °C). Cells were seeded in 24-well tissues culture plates (Falcon^®^) pre-filled with glass coverslips (Thermoscientific^®^) with a cell seeding concentration of 10,000 cells per well for the 24 h incubation time point and 1000 cells per well for the 72 h incubation time point. The 4 CCPM batches were diluted in PBS and used at a concentration of 0.1 mg/ml in culture medium. HUVECs were activated by TNFα (PromoKine^®^ recombinant human tumor necrosis factor alpha, *E. coli* derived 10 μl) at a concentration of 4 ng/mL for 4 h following a previously published protocol in [37]; before incubation with CCPM, TNFα-enriched medium was removed and fresh medium with CCPM was added. For the 72 h incubation time point at 37 °C, in addition to activated HUVEC (HUVEC^+^), quiescent HUVECs (HUVEC^−^) were also used. At the end of the experiment, coverslips were rinsed with PBS, and cells were fixed with 4% paraformaldehyde (PFA) for 20 min at room temperature (RT) and subjected to histology.

### Nanoparticles uptake assay under dynamic flow conditions

To validate the binding and uptake of control and cRGD-decorated CCPM under fluidic shear stress in vitro*,* only control and 5 mol% cRGD CCPM were used at a concentration of 0.1 mg/mL in medium. A total of 200,000 cells were seeded on 35-mm Petri dishes. Quiescent HUVECs were tested as additional negative control for the expression of integrin receptors. Petri dishes were placed into a customized parallel-wall flow chamber in a custom silicon tube perfusion system (standard silicon tubing, 0.76 mm inner diameter; Helixmark) [38]. Using a peristaltic pump (Gilson^®^), the flow rate was set with a speed of 0.20–0.25 ml/min for 10 min, followed by 5 min washing with PBS. Cells were then fixed with PFA and further processed for histology.

### Internalization studies with LysoTracker and flow cytometry

4T1 cells were cultured and incubated with non-targeted and cRGD-decorated CCPM for 24 h at 37 °C. Cells were then stained for 30 min (37 °C) for the lysosomial compartment with LysoTracker Green diluted in PBS, according to supplier’s protocol. For flow cytometry, 4T1 cells were seeded into 24-well plates (100,000 cells/ml per well) and incubated at 37 °C overnight. Cells were subsequentially incubated with CCPM for 24 h at 37 °C, washed, trypsinized, and cell suspension centrifuged at 1200 rpm for 5 min. After supernatant removal, the cell pellet was resuspended in ice-cold PBS and analyzed with the BD FACSCanto II Flow cytometry system (Becton, Dickinson and Company, USA).

### Immunofluorescence staining of ανβ3 and ανβ5 integrins

To evaluate the expression of ανβ3 and ανβ5 integrins, 4T1, HUVEC, and A431 cells were seeded in 24-well plates pre-filled with glass coverslips (Thermoscientific^®^) with a cell seeding concentration of 50,000 cells/well and incubated at 37 °C over night to allow cell attachment. For activating HUVEC, TNFα (PromoKine^®^ recombinant human tumor necrosis factor alpha, *E. coli* derived 10 μl) was used at a concentration of 4 ng/mL for 4 h; before starting the staining protocol, the TNFα-enriched medium was removed and cells were washed once with PBS. Following the fixation step with 4% PFA, cells were then treated with 200 µL of staining solution per well (*n* = 4 wells per staining per cell line) composed of ανβ3 antibody (Abcam^®^, UK) or ανβ5 antibody (Bioss antibodies^®^, USA) diluted resepctively 1:250 and 1:100 in 12% bovine serum albumin (BSA) (PAN Biontech^®^, Germany), according to the manufacturer’s instructions. Excess antibody was removed via three consecutive PBS washes, and cells were subsequetly incubated with the respective secondary antibodies, also diluted in 12% BSA (i.e., Cy3 anti-mouse (1:500), Cy3 anti-human (1:500; Dianova, Germany), DAPI (1:500; Merck^®^, Germany), and phalloidin (PromoKine^®^). Unbound antibodies were removed via PBS washes, coverslips were mounted with Mowiol 4–88 (Carl-Roth, Germany), and the glass covered to be stored at 4 °C.

### Histological analysis

Cells exposed to control and cRGD-CCPM were washed with PBS, fixed with 4% PFA for 20 min at RT, and washed again thrice with PBS. Primary antibodies (Abcam^®^ ab7166) were diluted in 12% bovine serum albumin (BSA) (PAN Biontech^®^, Germany) and applied for 1 h at RT. Excess antibody was removed via three consecutive PBS washes, and coverslips were subsequetly incubated with the respective secondary antibodies, also diluted in 12% BSA (Cy3-anti-rat (Dianova™ 115–165-166). Unbound antibodies were removed via PBS washes, coverslips were mounted with Mowiol 4–88 (Carl-Roth^®^, Germany) into glass slides, and the glass covered to be stored at 4 °C. For staining of the cytoskeleton and of nuclei, phalloidin (Promofluor^®^ 488 Phalloidin, PromoKine^®^) and DAPI (1:500; Merck^®^, Germany) were diluted in 12% bovine serum albumin (BSA) (PAN Biontech^®^, Germany) and applied for 1 h at RT. Coverslips were washed with PBS and mounted on object slides with Mowiol and stored at 4 °C.

### Fluorescent microscopy image analysis

For imaging cells incubated with CCPM and LysoTracker, all images were acquired with a fixed exposure times using a Zeiss® AxioImager M2 microscope (Carl Zeiss AG, Germany, 20 × objective, with a numerical aperture of 0.8) or an inverted Leica® DMI6000 B (Leica microsystems, Germany, 40 × objective with a numerical aperture of 0.95). For rhodamine and LysoTracker detection, fixed exposure times of 1500 ms and 1200 ms respectively, were used; for DAPI/Hoechst and phalloidin, exposure times were comprised in a range between 19 and 25 ms for the firsts, and between 50 and 100 ms for the latter. The image processing and quantification of area fraction percentage (AF %) for the fluorescence signals associated with rhodamine and and LysoTracker were performed using Axiovision LE and Leica Las AF software, respectively. For quantifying the uptake of control and cRGD decorated CCPM by target cells and by means for fluorescent microscopy imaging, at least 3 individual experiments including 3 cell-seeded coverslips per experiment were conducted, and 3 individual images per coverslip were acquired. For quantifying (i) the area fraction % of colocalization between rhodamine-CCPM and LysoTracker, (ii) the Pearson’s coefficient of colocalization, and (iii) the Manders’ colocalization coefficients for calculating the fraction of rhodamine-labeled CCPM overlapping with LysoTracker and the fraction of LysoTracker overlapping with rhodamine-labeled CCPM, the Fiji ImageJ plugin “JACoP” was used [39]. For imaging cells stained with α_ν_β_3_ and α_ν_β_5_ antibody, a Zeiss^®^ AxioImager M2 microscope with a 40 × objective (numerical aperture of 0.95) was used. Fixed exposure times of 1500 and 900 ms were used for detecting α_ν_β_3_ and α_ν_β_5_ antibody signal, respectively. The exposure times for DAPI and phalloidin were comprised in a range between 50 and 100 ms for the first, and between 200 and 250 ms for the latter. The image processing and quantification of area fraction percent (AF %) for the fluorescence signals associated with α_ν_β_3_ and α_ν_β_5_ antibody were performed using image processing package of ImageJ (Fiji).

### Two-photon laser scanning microscopy

To detect the 3D binding and uptake of targeted and non-targeted nanoparticles by 4T1 cells and assess their internalization process, two-photon laser scanning microscopy (TPLSM) was used.The FV1000MPE Multiphoton Microscopy System (Olympus^®^, Hamburg) was equipped with a Mai TaiDeepSee pulsed Ti:Sapphire laser and a 25 × water dipping objective with a numerical aperture of 0.95. The excitation wavelength was set to 800 nm with 15% power for the image acquisition. This wavelength allowed excitation of all probes. For the detection of fluorescence, signal one photo multipliertube per dye was used and the filters adjusted correspondingly to emission spectra. Image acquistion was executed with Kalman filtering, the following processing and 3D analysis were performed with the Imaris 7.4 software (Bitplane^®^, Zurich).

### Statistical analysis

All data are presented as mean ± standard error (SD). The number of experimental replicates is indicated in figure legends. Data were tested for levels of significance using a Kruskal–Wallis one-way ANOVA followed by Dunn’s multiple comparison correction test or by a two-tailed non-parametric Mann–Whitney’s test when only two groups were compared. Statistical analyses and data plotting were performed with GraphPad Prism^®^ 9.0 (GraphPad Software, Inc, San Diego, CA, USA). Levels of significance are indicated as follows: *p < 0.05; **p < 0.01; ***p < 0.001.

## Results and discussion

### cRGD-decorated CCPM display physicochemical properties comparable to non-modified CCPM

**Fig. 1:**
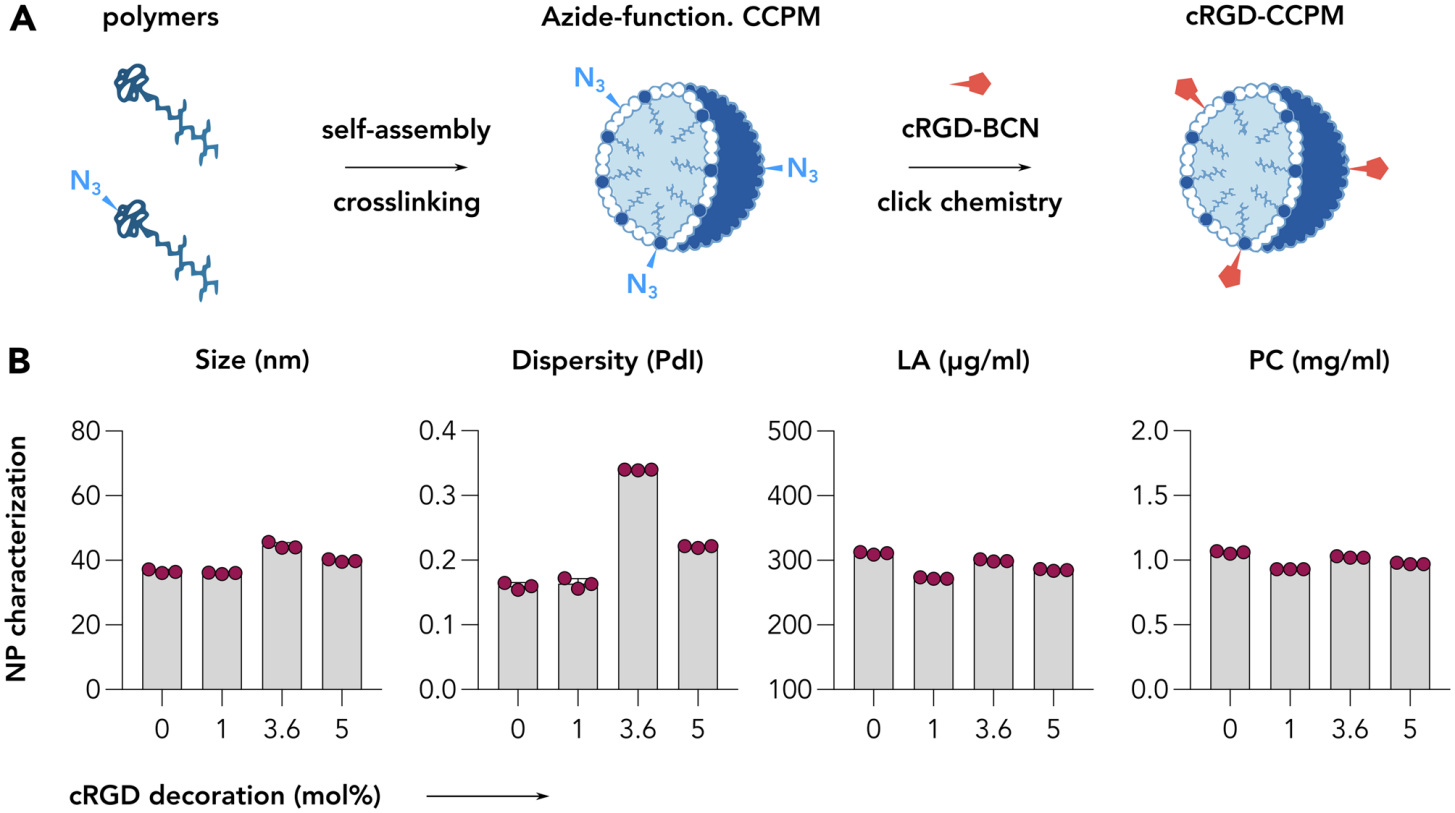
Synthesis and characterization of control and cRGD-decorated CCPM. **A** The schematic representation exemplifies the synthetic process of cRGD-CCPM, including the polymer units self-assembly and crosslinking, followed by the decoration of the CCPM by cRGD peptide via click chemistry. **B** The physicochemical characterization of CCPM including nanoparticle size (nm), dispersitiy (PdI), lactic acid content (LA; indirect indicator of polymer content), and polymer concentration (PC) indicates decorated and non-decorated CCPM to display a similar size of 35–40 nm, a polydispersity index comprised between 0.15 and 0.34, and a similar polymer content of approximately 1 mg/ml of polymer

By utilizing previously established methodologies [1, 4, 36], four CCPM nanoformulations with elevated amounts of cRGD were designed (Fig. 1A). The formulations contained 0, 1, 3.6, and 5 mol% of cRGD which corresponds to approximately 10, 36, and 50 cRGDfK units per nanoparticle [3, 40] Follow-up physicochemical characterization of the micelles revealed all four formulation to display a comparable size of 35–40 nm, polydispersity index between 0.15 and 0.34, and same polymer content that resulted in approximately 1 mg/ml of polymer (Fig. 1B).

### cRGD-decorated CCPM display time-, temperature-, and ligand density-dependent uptake in α_v_β_3_ integrin-expressing cell lines

A panel of four cell lines characterized by increasing expression levels of α_ν_β_3_ integrins (Fig. S1) was compiled: A431 (human epidermoid carcinoma cell line, α_ν_β_3_-integrin^low^ [41]), HUVEC (model of quiescent endothelial cells in blood vessels of healthy tissues, α_ν_β_3_-integrin^mid^; HUVEC^−^); TNFα-activated HUVEC (model of active and proliferative endothelial cells in angiogenic tumor blood vessels, α_ν_β_3_-integrin^high^; HUVEC^+^ [37]), and 4T1 (murine triple-negative breast cancer cells, α_ν_β_3_-integrin^high^). Comparatively, the four cell lines were also evaluated for their expression levels of α_ν_β_5_ integrins, with A431 and HUVEC^−^ to display a low expression, and the HUVEC^+^ and 4T1 to display a high expression (Fig. S2).

**Fig. 2:**
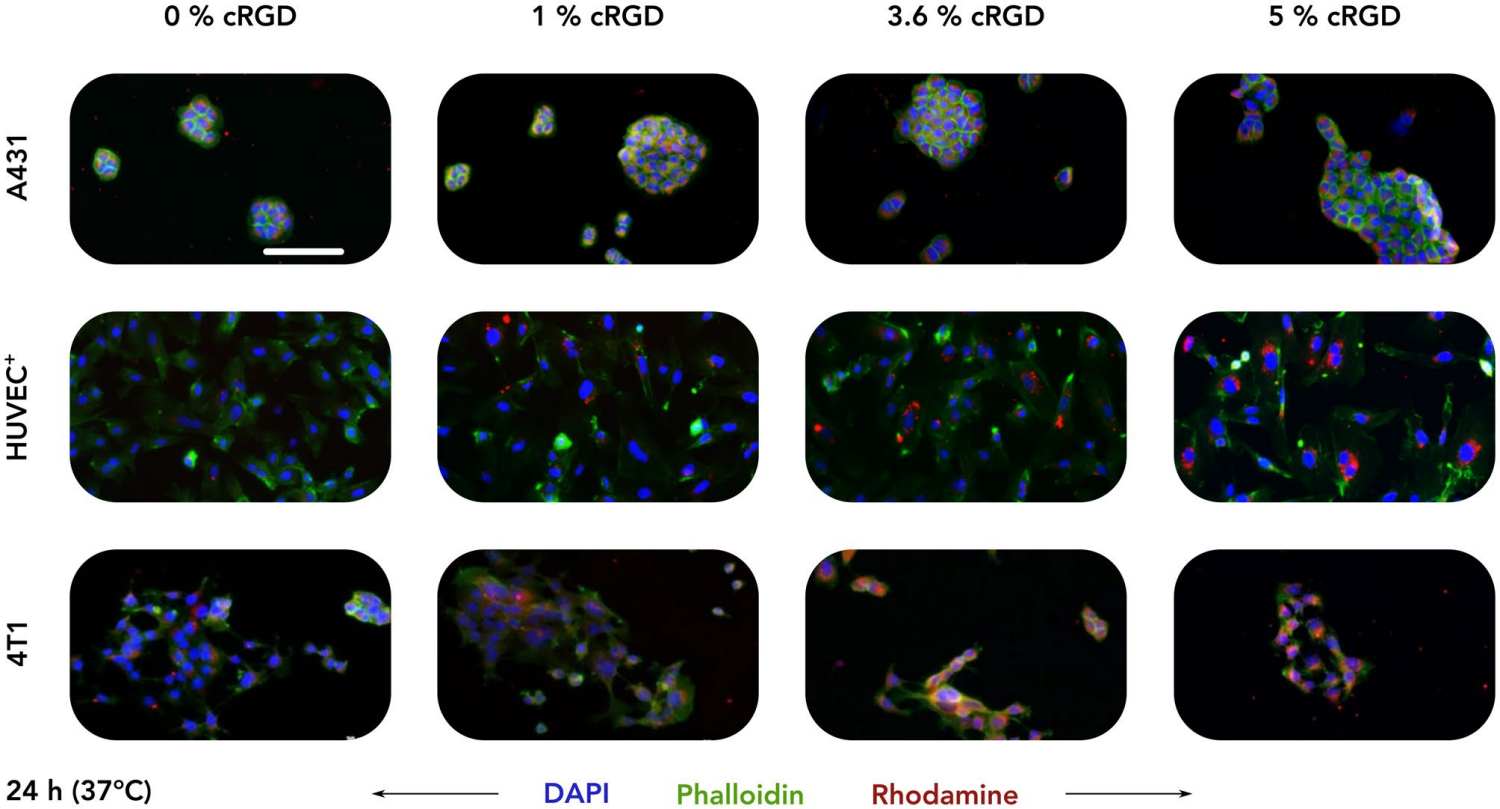
Uptake of control and cRGD-decorated CCPM after 24 h of incubation at 37 °C. Representative fluorescence microscopy images depict the uptake of rhodamine-labeled control and cRGD-targeted (1, 3.6, and 5 mol%) CCPM by A431 (human squamous carcinoma), TNFα-activated HUVEC (HUVEC^+^), and 4T1 (murine triple-negative breast cancer) cells 24 h post incubation, at 37 °C. All four nanoparticle formulations are taken up to a similar extent by A431 cells (α_v_β_3_-integrin^negative^ cell line), while increasing the cRGD-decoration density results in an enhanced uptake by HUVEC^+^ and 4T1 cells (α_v_β_3_-integrin.^positive^ cell lines). Color coding: DAPI (nuclei; blue), phalloidin (actin filaments; green), and rhodamine (micelles, red). Scale bar = 100 µm

**Fig. 3:**
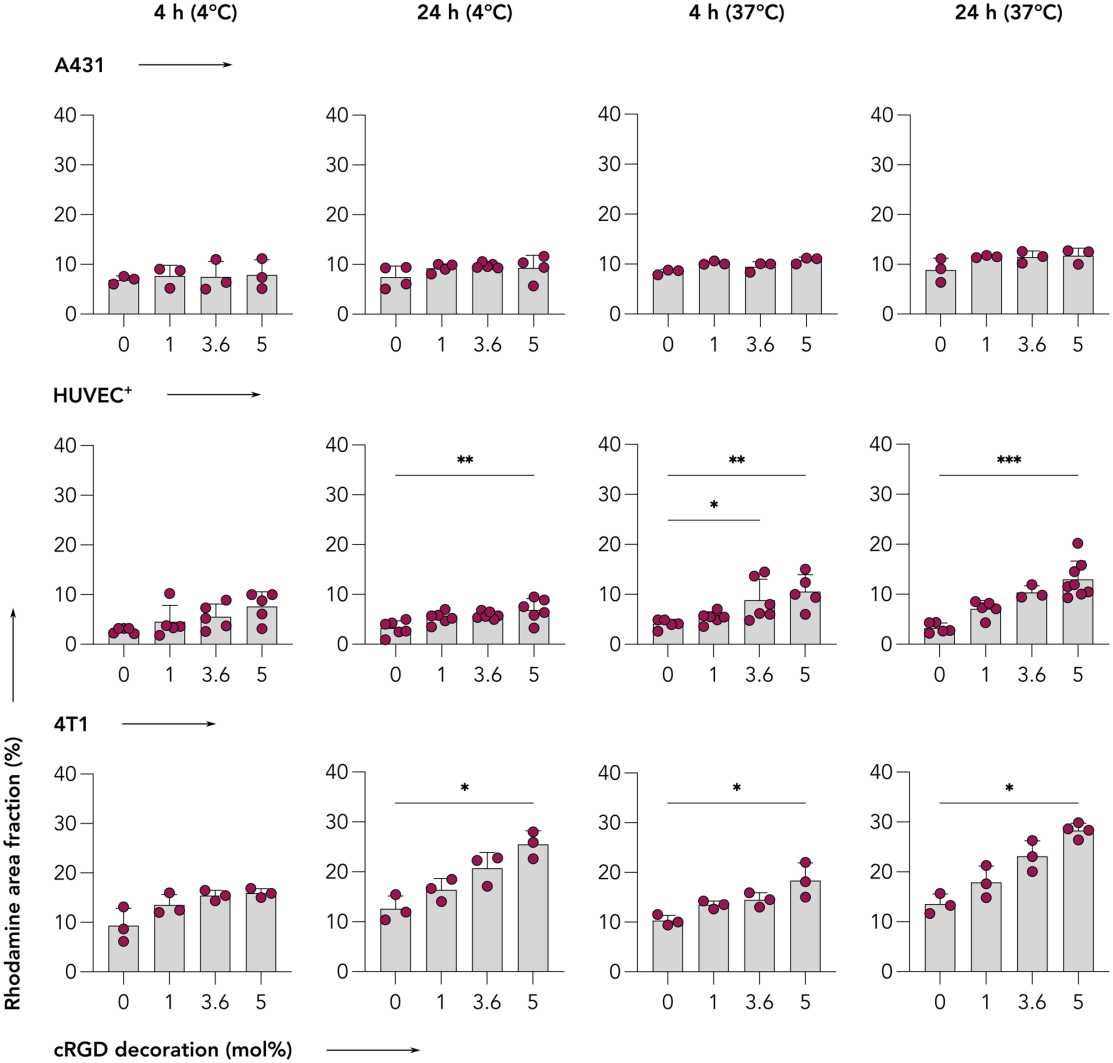
cRGD-decorated CCPM display time-, temperature-, and ligand density-dependent uptake in α_v_β_3_ integrin-expressing cells. Quantification of the uptake (i.e., rhodamine area fraction %) of control and cRGD-decorated micelles from fluorescence microscopy images (raw images presented in Figs. 1, S2, S3, and S4) reveals that all four nanoparticle formulations are taken up to similar extent by A431 cells (α_v_β_3_-integrin^negative^ cell line). Conversely, increasing the incubation time, incubation temperature, and cRGD density results in enhanced uptake by TNFα-activated HUVEC (HUVEC^+^) and 4T1 (α_v_β_3_-integrin.^positive^) cells. Data are presented as mean ± standard deviation of *n* = 3–7 biological replicates. Level of significance was assessed by a Kruskal–Wallis one-way ANOVA test, followed by Dunn’s multiple comparison correction test. *p*-values: * < 0.05; ** < 0.01, and *** < 0.001

Our first experiment involved the incubation of A431, HUVEC^+^, and 4T1 cell lines with all four cRGD-conjugated rhodamine-labeled CCPM formulations (0, 1, 3.6, and 5 mol% cRGD) for short periods of time, i.e., 4 and 24 h, at both 4 °C and 37 °C. In this case, 4 °C were selected to quantify the level of energy independent binding [42]. The degree of uptake was quantified as rhodamine area fraction % (AF%) in fluorescence microscopy images (Figs. 2, 3, S3, S4, and S5).

A431 cells displayed a low uptake of all four CCPM formulations at both temperatures of incubation, with only minor uptake deviations among the different groups. The uptake ranged between 6.9–7.9 AF% (4 h–4 °C), 7.5–9.8 AF% (24 h–4 °C), 8.5–10.8 AF% (4 h–37 °C), and 8.9–11.7 AF% (24 h–37 °C), exemplifying that in integrin-negative cells, longer incubation time and higher temperature contributed only very marginally to uptake enhancement. When comparing the different CCPM formulations within the same incubation time and temperature group, all 3 cRGD-decorated formulations displayed a similar uptake, with only a small and not statistically significant increase in uptake in comparison to the cRGD-free control formulation. Exemplarily, 5% cRGD decoration contributed to an additional 1.0, 1.8, 2.3, and 2.8 AF% for the 4 h–4 °C, 24 h–4 °C, 4 h–37 °C, and 24 h–37 °C group, respectively, in comparison to 0% decoration (Figs. 2, 3, and S3).

HUVEC^+^ cells, unlike A431 cells, displayed larger CCPM uptake deviations among the different incubation groups; a pattern that was expected considering the high α_v_β_3_ integrin expression by this cell line upon TNFα activation. The uptake spanned between 2.8–7.6 AF% (4 h–4 °C), 3.2–6.9 AF% (24 h–4 °C), 4.1–10.6 AF% (4 h–37 °C), and 3.2–13.0 AF% (24 h–37 °C), showing that higher temperature contributed to more efficient uptake. Comparing the uptake of the different CCPM formulations uptake within the same incubation time – temperature group, cellular internalization was consistently elevated in a step-wise manner, in line with cRGD density. For example, after 24 h of incubation at 37 °C, the CCPM uptake was 3.2, 7.1, 10.4, and 13.0 AF% for 0, 1, 3.6, and 5% cRGD, respectively. Overall, CCPM uptake was found to be significantly higher for 3.6 and 5% cRGD versus 0% cRGD at various incubation conditions (Figs. 2, 3, and S4), thus proving active uptake mediated by the integrin receptor and the additional uptake upon increasing cRGD on CCPM surface.

4T1 cells displayed the highest levels of internalization of CCPM in comparison to the other cell lines. Specifically, unlike the 6.9–11.7 and 2.8–13.0 AF% uptake-ranges observed by A431 and HUVEC^+^, 4T1 cells presented with values as high as 9.3–16.0 AF% (4 h–4 °C), 12.6–25.5 AF% (24 h–4 °C), 10.3–18.4 AF% (4 h–37 °C), and 13.5–28.3 AF% (24 h–37 °C). Incubation time was found to be essential for promoting CCPM internalization, while temperature increase less prominently contributed to uptake enhancement. As expected, and in line with the observations for HUVEC^+^, increasing the density of cRGD decoration corresponded with an increase in cellular uptake, with 5% cRGD conjugation resulting in significant uptake enhancement in comparison to control micelles for three out of four test condition (i.e., 24 h–4 °C, 4 h–37 °C, and 24 h–37 °C). The group with the largest uptake differences was the 24 h–37 °C group, where the CCPM uptake was 13.5, 17.9, 23.1, and 28.3 AF% for the 0, 1, 3.6, and 5% cRGD, respectively (Figs. 2, 3, and S5).

Together, these findings demonstrate that cRGD-decoration does not improve the uptake of CCPM by α_ν_β_3_-integrin^low^ cells. As compared to non-decorated CCPM, cRGD-decorated CCPM are taken up to a greater extent by α_ν_β_3_-integrin^high^ cells (4T1 and HUVEC^+^), in a time-, temperature-, and ligand density-dependent manner.

### Long incubation time points do not result in additional cRGD-CCPM uptake, while TNFα- activation enhances nanoparticle uptake by endothelial cells

**Fig. 4:**
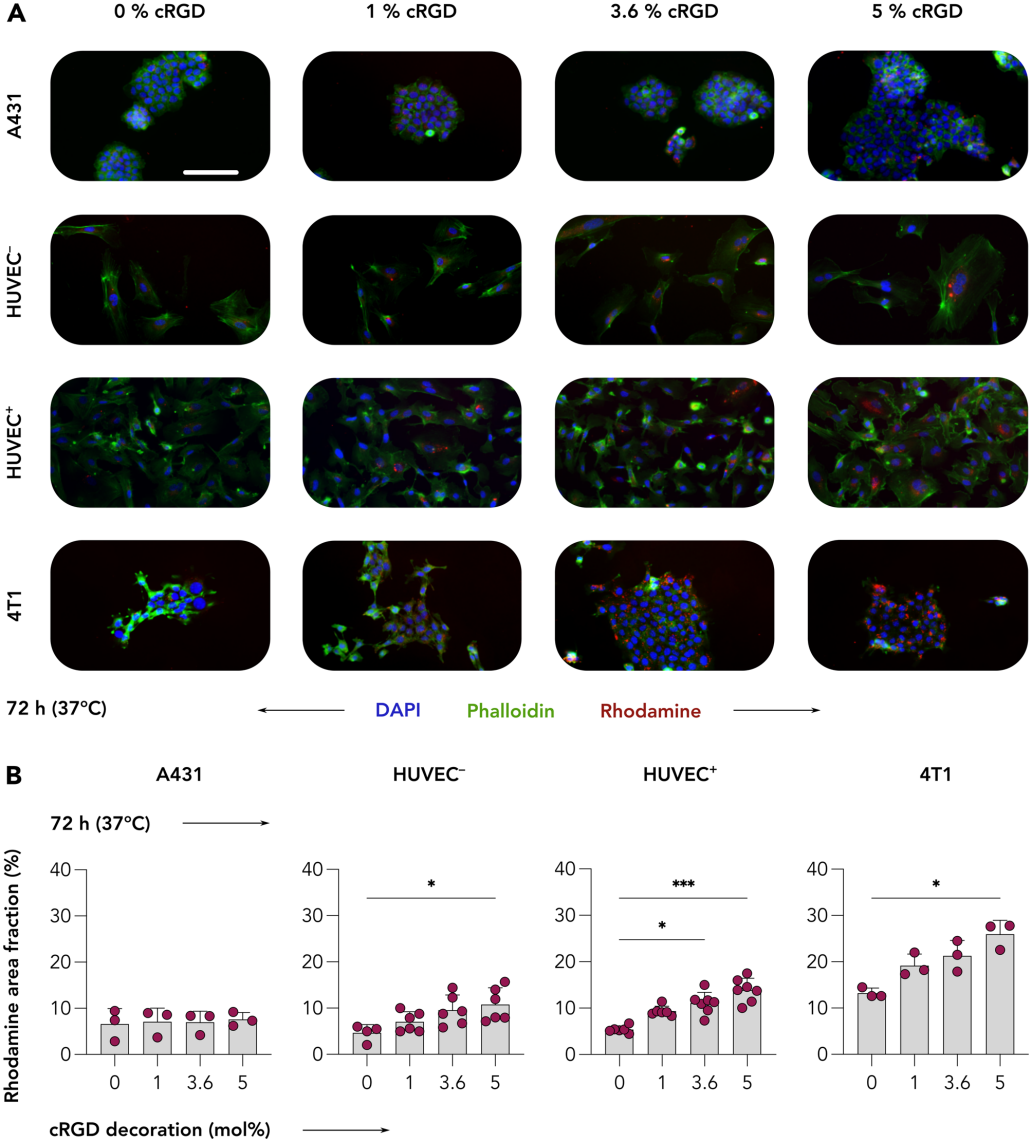
Uptake of control and cRGD-decorated CCPM by A431, HUVEC, and 4T1 cells after 72 h incubation. **A** Representative fluorescence microscopy images showing the uptake of control and cRGD-decorated micelles by A431, quiescent HUVEC (HUVEC^−^), TNFα-activated HUVEC (HUVEC^+^), and 4T1 cells at 72 h post incubation at 37 °C. Color coding: DAPI (nuclei, blue), phalloidin (actin filaments, green), and rhodamine (CCPM, red). Scale bar = 100 µm. **B** All four CCPM formulations are similarly taken up by A431 cells (α_v_β_3_-integrin^negative^), while increasing the cRGD-decoration density promotes uptake by HUVEC and 4T1 cells (i.e., all three α_v_β_3_-integrin.^positive^ cell lines). Importantly, TNFα-activated HUVEC display higher CCPM internalization in comparison to the non-activated cell line. Data are presented as mean ± SD. *N* = 3–7 biological replicates. Levels of significance were assessed by using a Kruskal–Wallis one-way ANOVA followed by Dunn’s multiple comparison correction test. *p < 0.05; **p < 0.01, and ***p < 0.001

The second experimental setup examined the uptake of the four CCPM formulations after a longer incubation time of 72 h. In this experiment, in addition to A431, HUVEC^+^, and 4T1 cells, also non-activated HUVECs (HUVEC^−^) were included. This addition allowed for investigating the effect of TNFα-mediated activation in CCPM uptake, because TNFα-based activation cause, among other effects, an increase in integrin expression by endothelial cells [43]. By comparing the uptake patterns between incubation for 24 h at 37 °C (Figs. 2 and 3) and incubation for 72 h at 37 °C (Figs. 4 and S6), it became evident that similar trends are observed with respect to the uptake of the four CCPM. Specifically, the uptake of all CCPM formulations by A431 cells was spanning within a short range, i.e., 6.6–7.6 AF%, with minor differences among the control and the cRGD-decorated formulations (Fig. 4). Conversely, after 72 h of incubation at 37 °C, HUVEC^+^ and 4T1 cells had efficiently internalized the CCPM that displayed the highest density of cRGD-decoration; a pattern also observable at shorter incubation times. In case of HUVEC^+^, the uptake was found to be 5.4, 9.3, 11.1, and 13.9 AF%, for 0, 1, 3.6, and 5% cRGD, respectively, with the values for 3.6 and 5% cRGD being significantly higher than for the cRGD-free control CCPM. Analogously, for 4T1 cells, the uptake was quantified at 13.2, 19.2, 21.3, and 26.0 AF% for the 0, 1, 3.6, and 5% cRGD, respectively, with the 5% cRGD CCPM displaying significantly higher uptake than control (Fig. 4).

In this experiment, a key point was to compare cRGD-targeted CCPM uptake between activated and non-activated HUVEC. For all four formulations, activated HUVEC displayed a higher degree of CCPM internalization in comparison to non-activated cells. The values of higher uptake of CCPM by HUVEC^+^ were quantified to range between 0.8 and 3.1 AF% as compared to the uptake by quiescent HUVEC. Specifically, the values of uptake of cRGD CCPM were of 5.4, 9.3. 11, and 14 for HUVEC^+^ for the 0, 1, 3.6, and 5% cRGD formulations respectively and of 4.6, 7.1, 9.6, and 10.8 AF% for the 0, 1, 3.6, and 5% cRGD for quiescent HUVEC (Fig. 4). Although these differences are not statistically significant when the values of AF% are compared head-to-head by cRGD decoration density for quiescent and activated, they are also not trivial, highlighting that TNFα-activated HUVECs have a propensity to internalize nanomaterial at higher extent. Such observation alludes to the more phagocytotic behavior of angiogenic/inflammatory endothelium versus the quiescent nature of mature non-inflammatory endothelium. This preferential uptake of nanomaterial by angiogenic/inflammatory endothelium versus quiescent endothelium has been already observed in vivo by us [13], where the angiogenic endothelium in 4T1-bearing animals displayed a 1.7 fold increase of cRGD nanoparticles uptake as compared to non-angiogenic endothelium.

### Dynamic cultivation conditions benefit cRGD-CCPM uptake by target cells

Our next experiment aimed at exploring how the parameter shear stress influences the recognition of the α_ν_β_3_ integrin by the various cRGD nanoformulations. To this end, we compared the internalization of 0 and 5% cRGD CCPM in A431, HUVEC^−^, HUVEC^+^, and 4T1 cells after a very short incubation time of 10 min at 37 °C under dynamic flow conditions, using a microfluidic chamber assay established previously [38]. This assay was used to study cRGD-CCPM internalization efficiency in a more realistic scenario as compared to the static set-up described above.

**Fig. 5:**
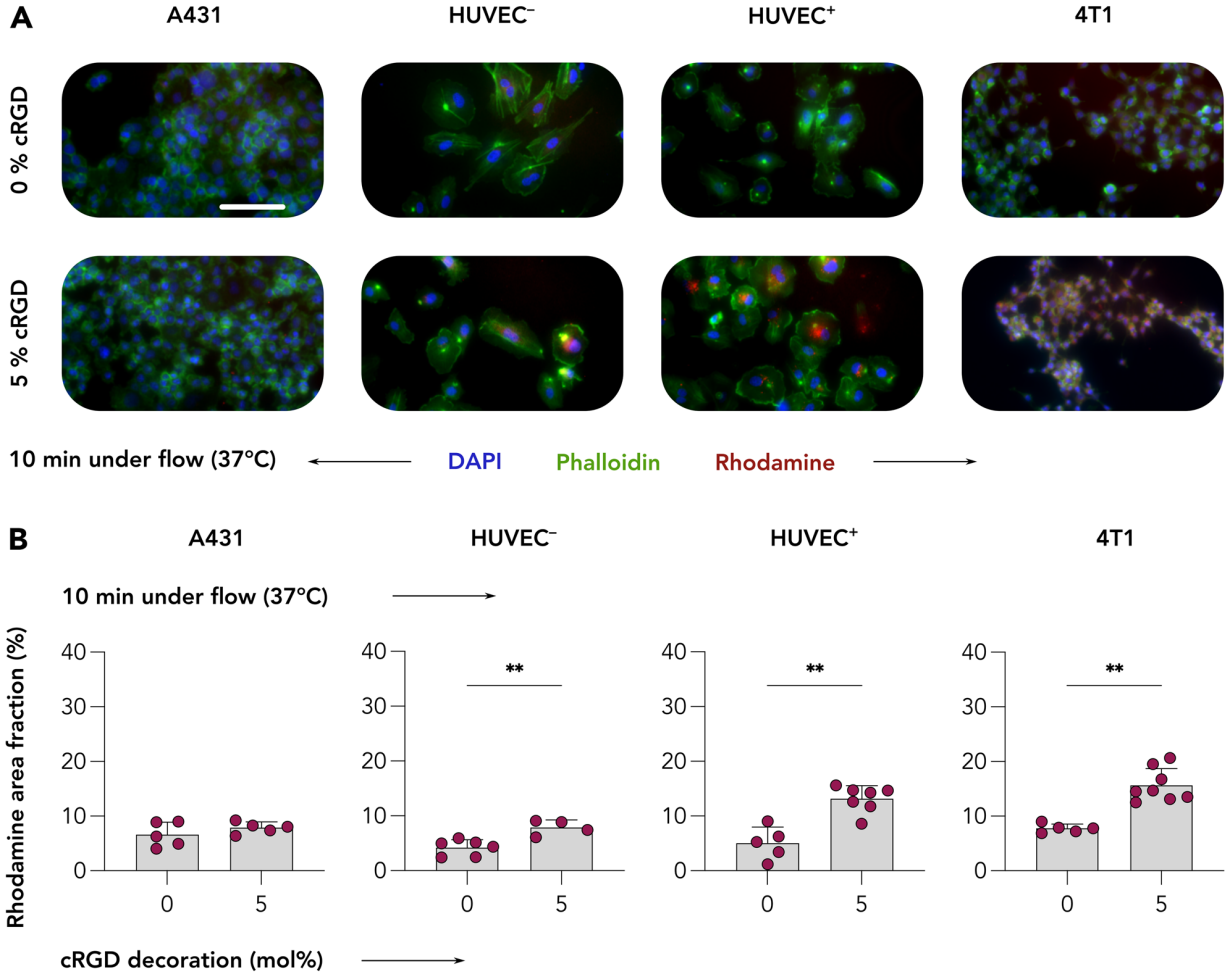
Uptake of control and cRGD-decorated CCPM upon 10 min of incubation under flow conditions. **A** Representative fluorescent microscopy images displaying A431, quiescent (HUVEC^−^), and TNFα-activated (HUVEC.^+^) HUVEC, as well as 4T1 cells following incubation with control and 5% cRGD-targeted CCPM under physiological fluid flow for 10 min at 37 °C. Color coding: DAPI (nuclei, blue), phalloidin (actin filaments, green), and rhodamine (CCPM, red). Scale bar = 100 µm. **B** Quantification of CCPM uptake (i.e., rhodamine area fraction %) shows a threefold increase in the uptake of cRGD-targeted CCPM by α_ν_β_3_ integrin-positive cells as compared to control CCPM, exemplifying that cRGD efficiently and rapidly mediates cellular uptake under shear stress conditions. Data are presented as mean ± SD. *N* = 5–8 biological replicates. Levels of significance were assessed by a Mann–Whitney’s non-parametric two-tailed. *p*-values: ** < 0.01

Dynamic incubation of all four cell types with control and 5% cRGD-decorated formulations showed similar uptake of the two formulations in A431 cells, but clearly higher uptake of the 5% cRGD formulation by HUVEC^−^, HUVEC^+^, and 4T1 cells (Figs. 5A and S7). Quantification of the images corroborated the qualitative observations, with for 0 and 5% cRGD CCPM uptake values of 6.6 and 7.9 AF% in A431 cells, 4.2 and 7.9 AF% in HUVEC^−^, 5.0 and 13.2 AF% in HUVEC^+^, and 7.8 and 15.7 AF% in 4T1 cells; all differences were found to be statistically significant (Fig. 5B). As for previous experiments conducted under static cultivation condition for 72 h at 37 °C, a key comparison was the one between quiescent HUVEC and HUVEC^+^ concerning the cRGD-CCPM uptake efficiency. The values of cRGD-CCPM uptake was of 7.9% for HUVEC^−^ and 13% for HUVEC^+^ respectively, with a *p* value of 0.0242 following a Mann–Whitney’s non-parametric, two-tailed *t*-test, thus confirming a statistically significant higher uptake of cRGD-CCPM by angiogenic/inflammatory endothelium as compared to quiescent endothelium.

This experiment under flow conditions clearly showed superior targeting ability of 5% cRGD CCPM in case of α_ν_β_3_ integrin expressing cells. Also, the rhodamine AF% quantified for HUVEC^+^ is slightly lower compared to the same recorded for 4T1, so the latter appears to be more keen to bind and internalize cRGD-CCPM. These results may likely be consequent to a combination of factors. First, a higher level of α_ν_β_3_ integrin expression by 4T1 cells compared to HUVEC^+^ cells and second, the higher metabolic rate of tumor cells that is associated to faster integrin internalization and recycling. Considering an eventual in vivo translation of the current study and taking the above results under flow conditions into account, it is reasonable to assume that cRGD-CCPM would bind more efficiently to angiogenic endothelial cells in tumor vasculature than to quiescent endothelial cells in healthy tissues. This is in line with various reports in the literature [38, 41, 44, 45].

Overall, the here presented experiments highlight efficient and rapid recognition of the α_ν_β_3_ integrin receptor by cRGD-decorated CCPM. This notion is in line with several in vitro observations, showing rapid and efficient integrin-mediated internalization already at 180 min post incubation with integrin positive cells [46]. In vivo observations obtained via, e.g., intravital microscopy showed significant uptake of cRGD-decorated lipid nanoparticles by α_v_ and β_3_ integrin subunit expressing immune cells already at very early time points post i.v. injection (i.e., 5, 10, 20 min) [13, 18].

### Analyzing CCPM nanoformulation internalization

**Fig. 6:**
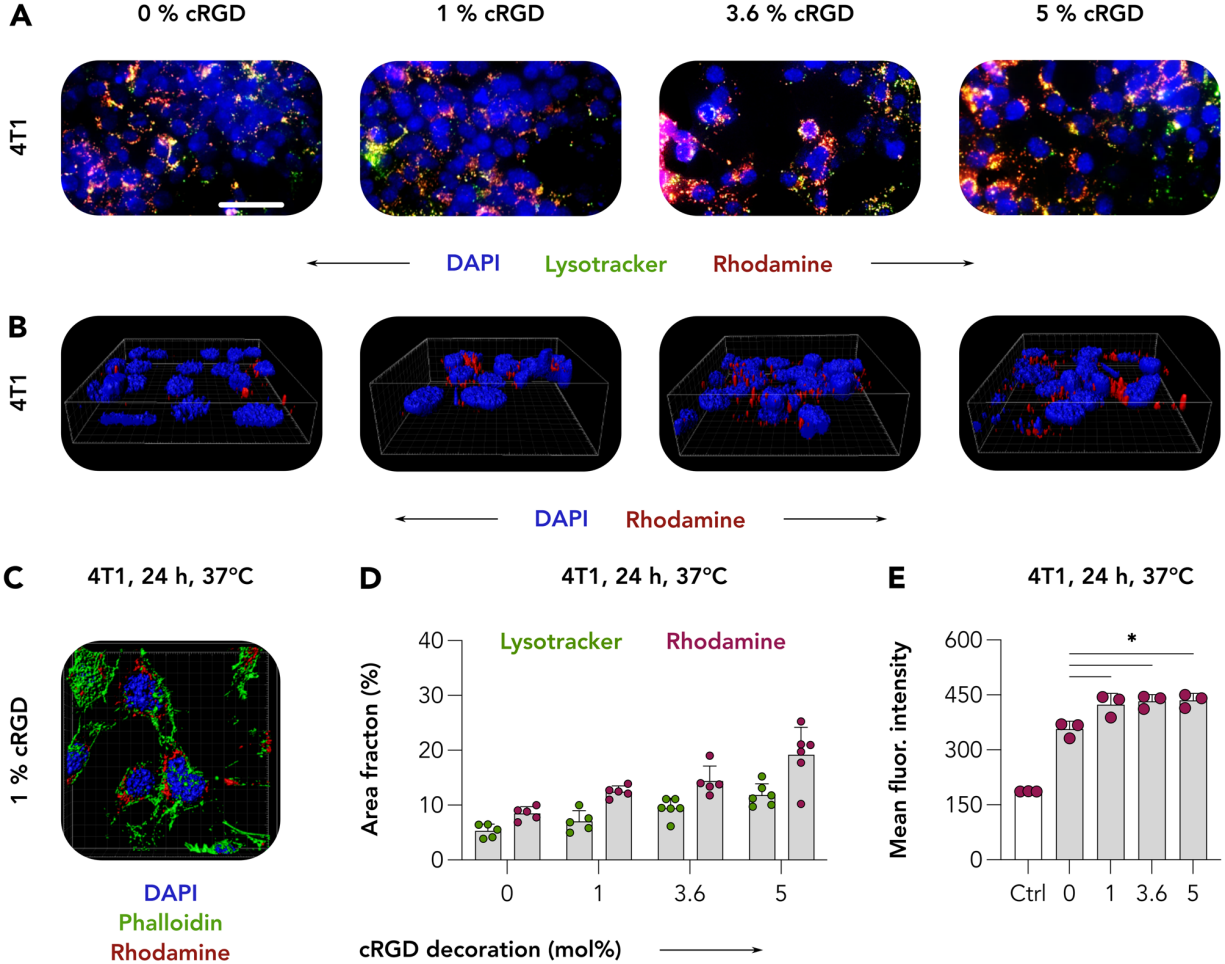
Internalization of cRGD-decorated CCPM. **A** 4T1 breast cancer cells were incubated with control and cRGD-CCPM for 24 h at 37 °C. LysoTracker was subquentially added to visualize the endosomal-lysosomal compartment. On live-cell imaging, the co-localization (yellow in merged images) of CCPM (red) with endosomes/lysosomes (green) was suggestive for CCPM internalization. Color coding: DAPI (nuclei, blue), LysoTracker (endo/lysosomes, green), and rhodamine (micelles, red). Scale bar = 100 µm. **B** and **C** Acquisition of 3D images via two-photon microscopy revealed cRGD-CCPM to be in the perinuclear region, further confirming the cRGD-CCPM internalization by 4T1 cells. Color coding: DAPI (nuclei, blue), phalloidin (actin filaments, green), and rhodamine (micelles, red). **D** Image analysis shows rhodamine and LysoTracker area fraction % to increase upon the increase of cRGD-decoration density. **E** Quantification of flow cytometry analysis of micelle binding/uptake by 4T1 cells displays a statistically significant increase in uptake of cRGD-conjugated CCPM in comparison to cRGD-free CCPM. Data are presented as mean ± SD of *n* = 3 biological replicates; levels of significance were assessed by using a one-way ANOVA followed by Tukey’s correction. *p*-values: * < 0.05

Our next experiments aimed at unraveling the contribution of integrin-receptor targeting to the internalization of cRGD-conjugated CCPM by α_ν_β_3_-integrin^high^ 4T1 cancer cells. This was done because integrins – in addition to being popular membrane targets on several different cell types – are known to be efficient internalizers, to engage in endo/phagocytosis pathways, and to be rapidly recycled [47, 48]. For this purpose, upon incubation with control and cRGD-decorated CCPM for 24 h at 37 °C, 4T1 cells were stained with LysoTracker to visualize endosomal-lysosomal trafficking. On live-cell imaging, the co-localization of CCPM (red) with endosomes/lysosomes (green) produced a yellow/orange fluorescent signal in merged images, suggestive for CCPM cellular internalization (Figs. 6A and S8). In addition to this, the acquisition of 3D multiphoton microscopy images revealed the rhodamine-labeled nanoformulations, particularly the 5% cRGD-decorated CCPM, to localize in close proximity to cell nuclei (Fig. 6B), further corroborating the notion of efficient integrin-mediated CCPM cellular internalization. Additional images acquired via multiphoton microscopy in which 4T1 cells were additionally stained with phalloidin to visualize actin filaments (Fig. 6C, green), clearly displayed red clusters of cRGD-modified CCPM to localize in the cytosolic perinuclear space. These observations were in line with others in literature, e.g., using 5% cRGD nanocarriers in MDA-MB-231 cells [49].

The quantification of rhodamine AF% on fluorescence live cell imaging (Fig. 6D) confirmed the preceding data obtained using fluorescence microscopy in fixed cells, with cRGD-CCPM binding being positively affected by increasing amounts of cRGD decoration. Of note, the quantification of LysoTracker AF% also follows a similar increasing trend as rhodamine nanoparticles, with more LysoTracker signal being quantified upon increased uptake of nanoparticles. Because recent evidences show the ability of cells to increase the number of lysosomes in reponse to degradative or energetic needs, it is reasonable to speculate that increased uptake of nanomaterial may be reflected into an increased number of endosomes and lysosomes [50]. Additional quantification of the colocalization rate between rhodamine-labeled CCPM and LysoTracker revealed that decoration with cRGD translates into higher values of colocalization of rhodamine-labeled CCPM and LysoTracker as compared to undecorated CCPM. Among the decorated formulations, we found the best correlation coefficient between LysoTracker and rhodamine for the 1 mol% cRGD (Pearson’s correlation coefficient of 0.805) and the lowest for the 5 mol% cRGD (Pearson’s correlation coefficient of 0.669). In line with this observation, we observed that the majority of lysosomes to overlap with rhodamine-CCPM, whereas not all rhodamine-CCPM signal overlaps with lysosomes for the 5 mol% cRGD formulation, which is suggestive for mechanistic saturation (Fig. S8). Higher-resolution imaging modalities, such as super-resolution STED (for live or fixed cells) or electron microscopy (for fixed cells only), combined with more specific endosomes/lysosome-stainings (e.g., EEA1 and Rab5 for early endosomes, Rab7 for late endosomes and LAMP-1 for lysosomes) would be suitable tools to further dissect the intracellular fate of cRGD-CCPM and to study the dynamics of the lysosomial compartment upon increased uptake of CCPM.

Finally, we also studied the internalization of cRGD-decorated CCPM by flow cytometry. This analysis confirmed that the association of cRGD-CCPM (1, 3.6, and 5 mol%) with 4T1 cells upon incubation for 24 h at 37 °C was significantly higher than that of cRGD-free control CCPM. The mean fluorescence intensity values (MFI) of 357, 424, 433, and 435 were obtained for the 0, 1, 3.6, and 5 mol% cRGD CCPM, respectively (Fig. 6E).

### Identifying the optimal cRGD decoration density for follow-up in vivo studies

Cyclic RGD is among the most widely studied targeting ligand and it has demonstrated strong affinities for α_v_β_3_ integrin, a key receptor expressed by multiple cell populations including among others, cancer cells of the skin and of the breast, endothelial cells, and neutrophils. Surface functionalization of nanoparticles with targeting ligands such as cRGD has shown significant advantages in preclinical cancer nanotherapy studies [51, 52]. However, in-depth cellular examination already revealed that RGD-targeting induced nanoparticle association with tumor vasculature while marginally reaching the tumor interstitium [29, 53]. In addition, in the bloodstream, targeting moieties present on the nanoparticle surface may cause elevated recognition by the mononuclear phagocyte system (MPS) and thereby compromise nanoparticle PK characteristics and tumor accumulation [13, 35, 49]. Thus, it is key to identify the right decoration density to balance the increased uptake efficacy and good PK prolifes for in vivo applications.

**Fig. 7:**
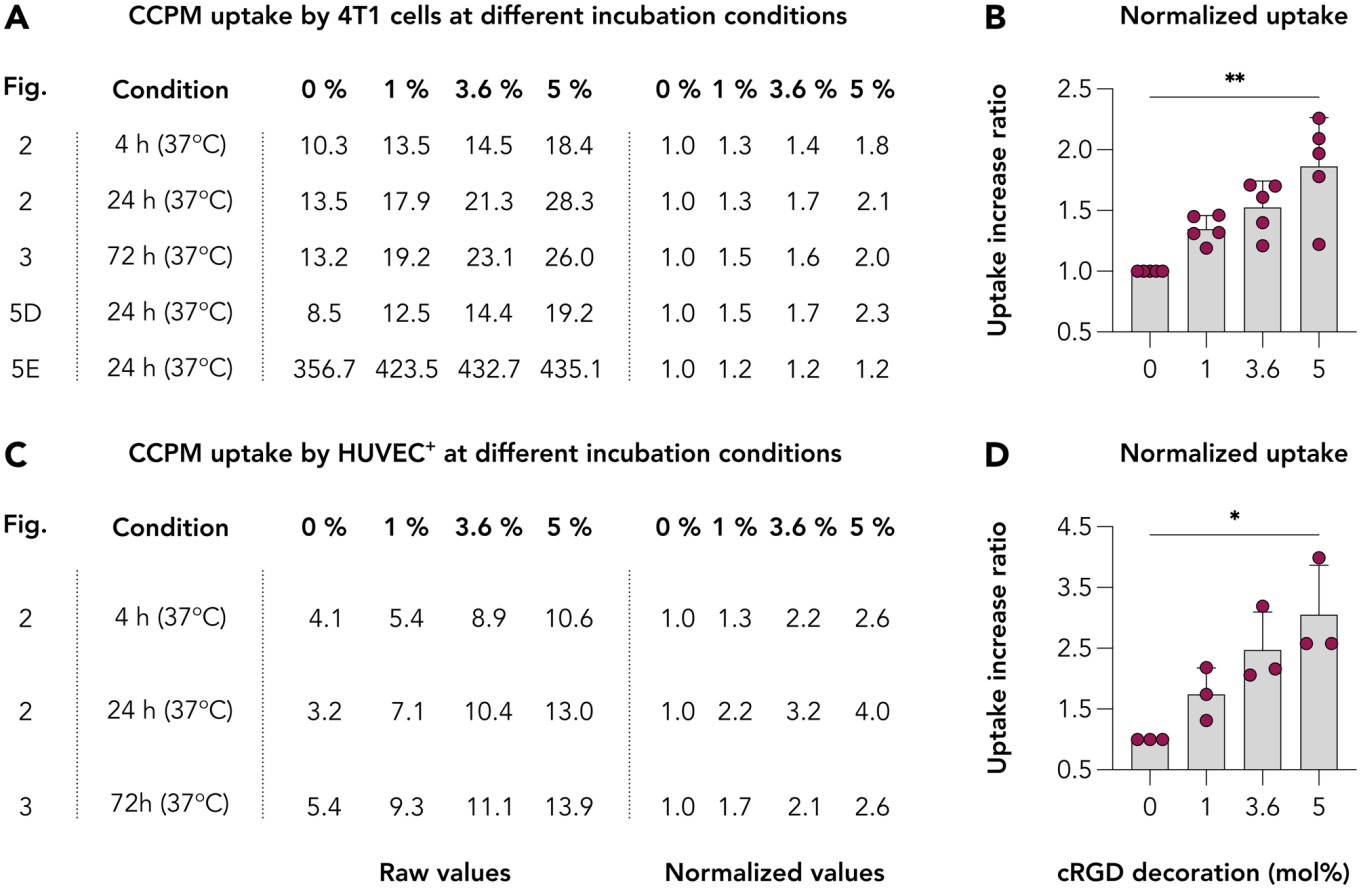
Quantitative comparison of the uptake of control and cRGD-decorated CCPM by α_ν_β_3_-integrin^high^ cell lines. **A** and **B** Quantification of the uptake of control and cRGD-decorated CCPM by 4T1 cells. **C** and **D** Quantification of the uptake of control and cRGD-decorated CCPM by HUVEC.^+^. Despite the elevated uptake of the cRGD-decorated CCPM in comparison to control CCPM by both cell lines, the uptake is not proportional to the decoration density. In other words, both 3.6 and 5 mol% decoration does not quantitatively improve the CCPM uptake in comparison to the 1 mol% cRGD decoration. This observation denotes that higher and higher decoration densities do not result in “inifinite” increases with respect to the formulation binding or uptake

For identifying the benefit of cRGD decoration, we quantitively compared the binding and internalization of the various CCPM formulations by both the α_ν_β_3_-integrin^high^ cell types used (4T1, TNFα-activated HUVEC) and at all available experiments. For 4T1 cell line, five experiments involved incubation of these cells with all four CCPM formulations, including incubation assay for 4, 24, and 72 h at 37 °C and evaluation via fluorescence microscopy (Figs. 3 and 4), internalization assay for 24 h at 37 °C and evaluation via multiphoton microscopy (Fig. 6D), and finally quantification of the uptake extent after incubation for 24 h at 37 °C via flow cytometry (Fig. 6E). For HUVEC^+^, three experiments entailed the incubation of the cells with all four CCPM formulations, i.e., incubation assay for 4, 24, and 72 h at 37 °C and evaluation via fluorescence microscopy (Figs. 3 and 4). By comparing the binding/uptake values for each nanoformulation at these experiments, and by normalizing these values based on the binding/uptake of control CCPM (0 mol% cRGD) for each individual experiment, we displayed the enhanced uptake ratio for each formulation for each different experiment (Fig. 7). For 4T1 cells, the uptake increase ratio ranged between 1.2–1.5, 1.2–1.7, and 1.2–2.3 for the 1, 3.6, and 5% cRGD CCPM (Fig. 7A and B). Considering the additional 20–50% uptake due to the 1% cRGD decoration, from a theoretical and proportional point of view, the 3.6 and 5% cRGD CCPM should result into an additional uptake comprised between 72–180% and 100–250%, respectively. Analysis of our data set reveals instead that a decoration density of 3.6 and 5% cRGD resulted in an additional uptake between 20–70% and 20–130% by 4T1 cells as compared to control CCPM. For HUVEC^+^ cells, the uptake increase ratio ranged between 1.3–2.2, 2.1–3.2, and 2.6–4.0 for the 1, 3.6, and 5% cRGD CCPM (Fig. 7C and D). Similarly to the abovementioned calculations, the additional uptake due to the 1% cRGD decoration was of 30–120%, and it should theoretically be within the range of 108–432%, and 150–600%, for the 3.6 and 5% cRGD CCPM, respectively, if the increase in the cRGD would be accompanied by a proportional increase in uptake by target cells. However, the additional increase ranged at the lower end of the theoretical values, i.e., 110–220% and 160–300% for the 3.6 and 5% cRGD CCPM, respectively. Such observation indicates that increasing the decoration density does not necessarily contribute to a proportional increase in binding and/or uptake. Besides by particle size and charge, several studies have evidenced that surface decoration with targeting ligands modulates BD profiles [35, 49, 54]. For example, high cRGD decoration density can shift the accumulation of nanoparticles to the RES system and reduce the nanoparticle availability for targeting purposes, thus, requiring an additional PEG-shielding for ameliorating this effect [49]. In addition, high amounts of ligand decoration may promote unwanted protein corona formation, with opsonization in the blood and uptake by phagocytes in liver and spleen [35]. Finally, high concentrations of cRGD might negatively modulate the downstream signaling events, such as a loss in cadherin-dependent intercellular contacts [55]. Of note, also the clinical trialing of cRGD in the form of a cRGD-decorated silica nanoparticles was done via a low functionalization as only 6–7 cRGD molecules were added per particle. This choice was specifically done for maintaining a small hydrodynamic size that would not significantly shift the BD profile of the evaluated diagnostic nanoparticle [56]. In addition, it is important to state that CCPM is a formulation with negligible formation of protein corona [57], therefore low decoration densities might be suitable for maintaining this desirable feature for prolonged circulation and efficient tumor targeting. In addition, we [13, 18, 31] and others [23, 46, 53, 58] have observed that low decoration densities (i.e., 1 mol% or lower) are sufficient for significantly altering the in vivo behavior between ligand-decorated and non-decorated analogs. Of note, and considering our goal to evaluate this drug delivery system in vivo, the used nanoformulation has already undergone extensive investigation regarding shelf stability [59], and serum stability and protein binding examined via asymmetrical flow field-flow fractionation [57]. Regarding the latter, the crosslinking principle has assured the serum stability as can be illustrated by the extensive circulation upon intravenous administration, both preclinically [4, 6] and clinically [7, 8].

Taken together, by comparing the in vitro uptake patterns in the α_ν_β_3_-integrin^high^ cell lines, and considering previous observations from in vivo and clinical set-ups where cRGD decorations were used, we suggest that low decoration densities (in the range of 1 mol%) are sufficient to beneficially alter the targeting behavior of the formulations, but not too high for causing deleterious effects on their in vivo behavior (i.e., improper PK and extensive deposition in clearance organs).

## Summarizing discussion

The present study aimed at expanding our understanding of the effect of ligand decoration densities on the active targeting properties of clinical-stage platform based on core-crosslinked polymeric micelles (CCPM). In this regard, we evaluated the uptake of rhodamine-labeled CCPM decorated with increasing cRGD densities (1, 3.6, and 5 mol%) against α_ν_β_3_-integrin^high^ (i.e., 4T1 breast cancer and TNFα-activated HUVEC), α_ν_β_3_-integrin^low^ cells (i.e., A431 epidermoid carcinoma cell line), and α_ν_β_3_-integrin^intermediate^ cells (non-activated HUVEC).

Our results show that (i) the uptake of cRGD CCPM is time- and temperature-dependent, with the optimal uptake condition registered at 24 h post incubation at 37 °C; that (ii) the uptake efficacy of cRGD CCPM is higher in α_ν_β_3_-integrin^high^ cells, both under static and dynamic cultivation conditions, thereby confirming the functionality and additional benefit of the ligand; that (iii) cRGD CCPMs are more efficiently internalized by target cells as compared to control CCPM and localize in proximity to cell nuclei; also, internalized particles co-localize with lysosomes, thus highlighting the additional efficacy of exploiting α_ν_β_3_ integrin-based internalization strategies; and that (iv) the increase in cRGD decoration density does not result in a proportional increase in the uptake of functionalized CCPM, suggesting that relatively low decoration densities in the range of 1 mol% cRGD may already be sufficient for evoking a change in CCPM targeting and uptake in vivo.

Concerning the selection of a relatively low cRGD mol% decoration for eventual in vivo experimentation, it should be kept in mind that the extensive presence of targeting ligands may affect the PK and BD profile of nanonomedicines. Besides by particle size and charge, several studies have evidenced that surface decoration with targeting ligands modulates BD profiles [35, 49, 54]. For example, high cRGD decoration density can shift the accumulation of nanoparticles to the RES system and reduce the nanoparticle availability for targeting purposes, thus, requiring an additional PEG-shielding for ameliorating this effect [49]. In addition, high amounts of ligand decoration may promote unwanted protein corona formation, with opsonization in the blood and uptake by phagocytes in liver and spleen [35]. Finally, high concentrations of cRGD might negatively modulate the downstream signaling events, such as a loss in cadherin-dependent intercellular contacts [55].

## Conclusion

Using static and dynamic cultivation studies, we profiled the cellular uptake of actively targeted core-crosslinked polymeric micelles in vitro. Our results showed benefit of active targeting with respect to the internalization of CCPM by target cells. Comparing the three cRGD-decorated nanoformulations tested, the highest decoration density (5 mol % cRGD) resulted in the highest uptake and internalization rate of CCPM by targeted cells, under all experimental conditions tested. However, in depth analysis evidenced that increasing the decoration density does not necessarily contribute to a proportional increase in binding and/or uptake. Taken into account that it is key to identify the right decoration density, we suggest that low decoration densities are capable of improving the cell targeting of formulations, but not too high to cause detrimental effects on their in vivo behavior.

## Supplementary information

Below is the link to the electronic supplementary material.

Supplementary file1 (DOCX 6541 KB)

## Data Availability

Raw data are available by the authors upon request.
