# Supplementary material for: Profiling target engagement and cellular uptake of cRGD-decorated clinical-stage core-crosslinked polymeric micelles
Source: Drug Deliv Transl Res. 2022 Jul 11;13(5):1195–211. doi: 10.1007/s13346-022-01204-8 (PMC10102119; doi:10.1007/s13346-022-01204-8)
Supplement: Supplementary file 1 — Supplementary file1 (DOCX 6541 KB) [file 13346_2022_1204_MOESM1_ESM.docx]

**SUPPLEMENTARY MATERIAL**

**Profiling target engagement and cellular uptake of cRGD-decorated**

**clinical-stage core-crosslinked polymeric micelles**

Federica De Lorenzi^1^, Larissa Yokota Rizzo^1^, Rasika Daware^1^, Alessandro Motta^1^,

Maike Baues^1^, Matthias Bartneck^2^, Michael Vogt^3^, Marc van Zandvoort^4,5^, Leonard Kaps^6,7^,

Qizhi Hu^8^, Marielle Thewissen^8^, Luca Casettari^9^, Cristianne J.F. Rijcken^8^,

Fabian Kiessling^10,11^, Alexandros Marios Sofias^1,12,13^, Twan Lammers^1,*^

1. Department of Nanomedicine and Theranostics, Institute for Experimental Molecular Imaging (ExMI), RWTH Aachen University Clinic, Aachen, Germany
2. Clinic for Gastroenterology, Metabolic Diseases and Internal Intensive Care Medicine (Internal Medicine III), RWTH Aachen University Clinic, Aachen, Germany
3. Interdisciplinary Center for Clinical Research (IZKF), RWTH Aachen University Clinic, Aachen, Germany
4. Institute for Molecular Cardiovascular Research (IMCAR), RWTH Aachen University Clinic, Aachen, Germany
5. Department of Genetics and Cell Biology, School for Cardiovascular Diseases (CARIM), School for Oncology and Reproduction (GROW), School for Mental Health and Neuroscience (MHeNS), Department of Genetics and Cell Biology, Maastricht University, Maastricht, the Netherlands
6. Department of Medicine, University Medical Center Mainz, Mainz, Germany
7. Institute of Translational Immunology and Research Center for Immune Therapy, University Medical Center Mainz, Mainz, Germany
8. Cristal Therapeutics, Maastricht, The Netherlands
9. Department of Biomolecular Sciences, University of Urbino Carlo Bo, Urbino, Italy
10. Institute for Experimental Molecular Imaging (ExMI), RWTH Aachen University Clinic, Aachen, Germany
11. Fraunhofer Institute for Digital Medicine MEVIS, Bremen, Germany
12. University Hospital Aachen, Mildred Scheel School of Oncology (MSSO), Center for Integrated Oncology Aachen Bonn Cologne Düsseldorf (CIO^ABCD^), Aachen, Germany
13. Norwegian University of Science and Technology (NTNU), Department of Circulation and Medical Imaging, Faculty of Medicine and Health Sciences, Trondheim, Norway

* Corresponding author: T. Lammers ([tlammers@ukaachen.de](mailto:tlammers@ukaachen.de))

Supplementary Figure 1


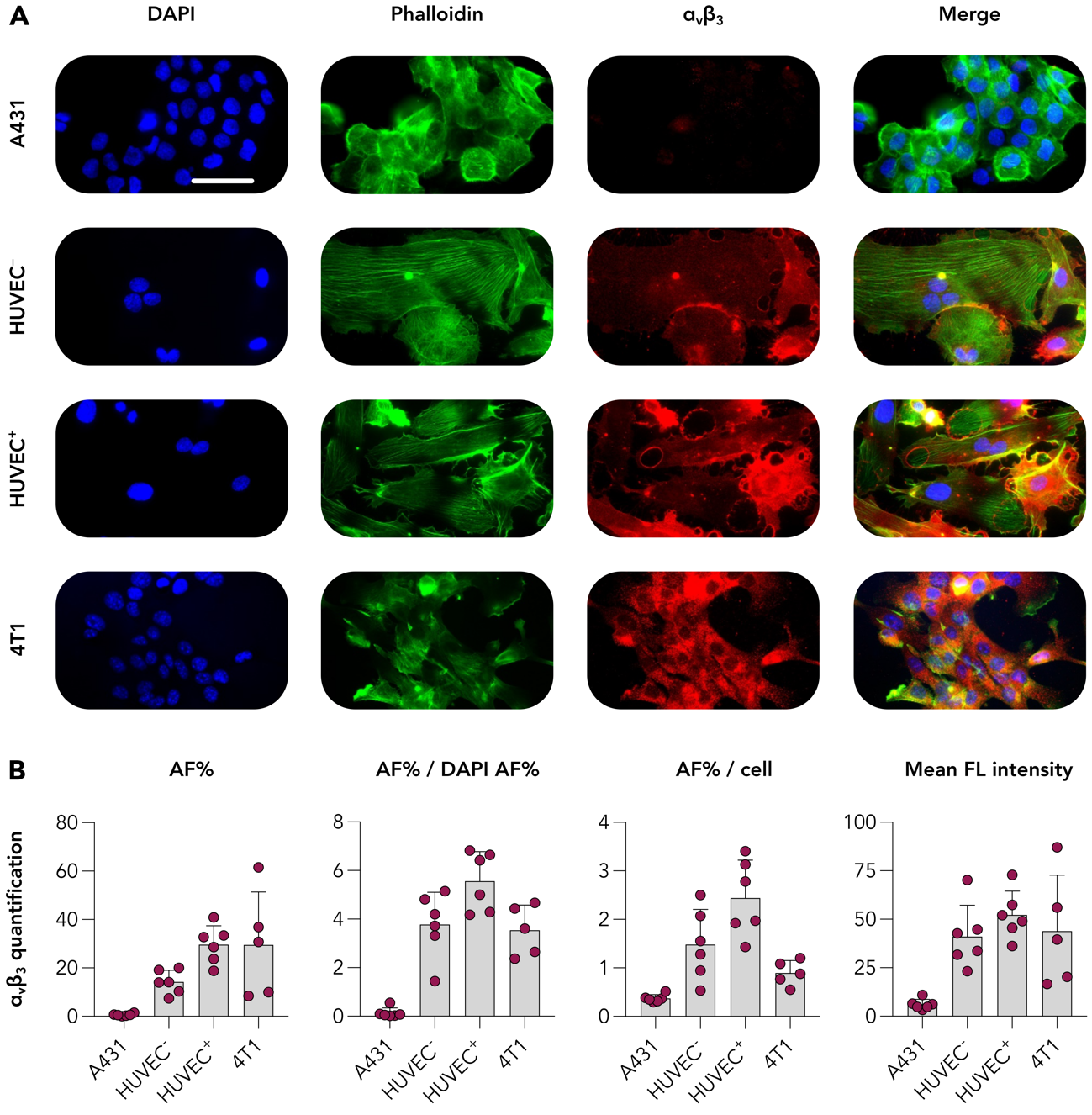


**Figure S1. Visualization of α_v_β_3_ integrin expression levels by A431, quiescent HUVEC (HUVEC^-^), activated HUVEC (HUVEC^+^), and 4T1.** Based on the antibody staining against the α_v_ and β_3_ integrin sub-units, the A431 is characterized as α_v_β_3_-integrin^low^, the HUVEC^-^ as α_v_β_3_-integrin^intermediate^, and the HUVEC^+^ and 4T1 as α_v_β_3_-integrin^high^. α_v_β_3_ is the major target of cRGDfK peptide. Color coding: DAPI in blue, phalloidin in green, α_v_β_3_ integrin in red. Scale bar = 60 μm.

Supplementary Figure 2


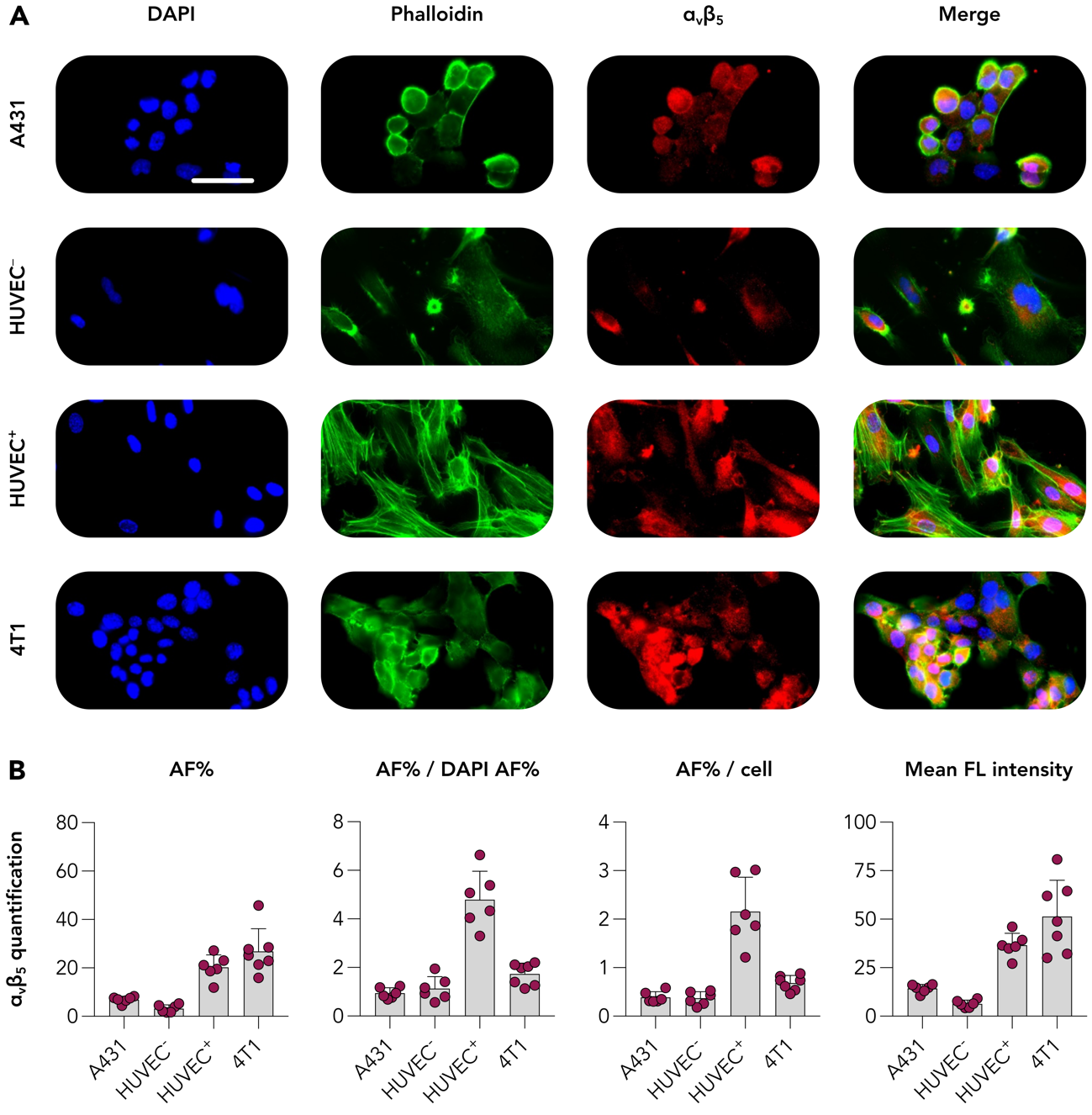


**Figure S2. Visualization of α_v_β_5_ integrin expression levels by A431, quiescent HUVEC (HUVEC^-^), activated HUVEC (HUVEC^+^), and 4T1.** Based on the staining against the α_v_ and β_5_ integrin sub-units, the A431 and HUVEC^-^ are characterized as α_v_β_5_-integrin^low^, and the HUVEC^+^ and 4T1 as α_v_β_3_-integrin^high^. α_v_β_5_ is a supplementary target of cRGDfK peptide. Color coding: DAPI in blue, phalloidin in green, α_v_β_5_ integrin in red. Scale bar = 60 μm.

Supplementary Figure 3


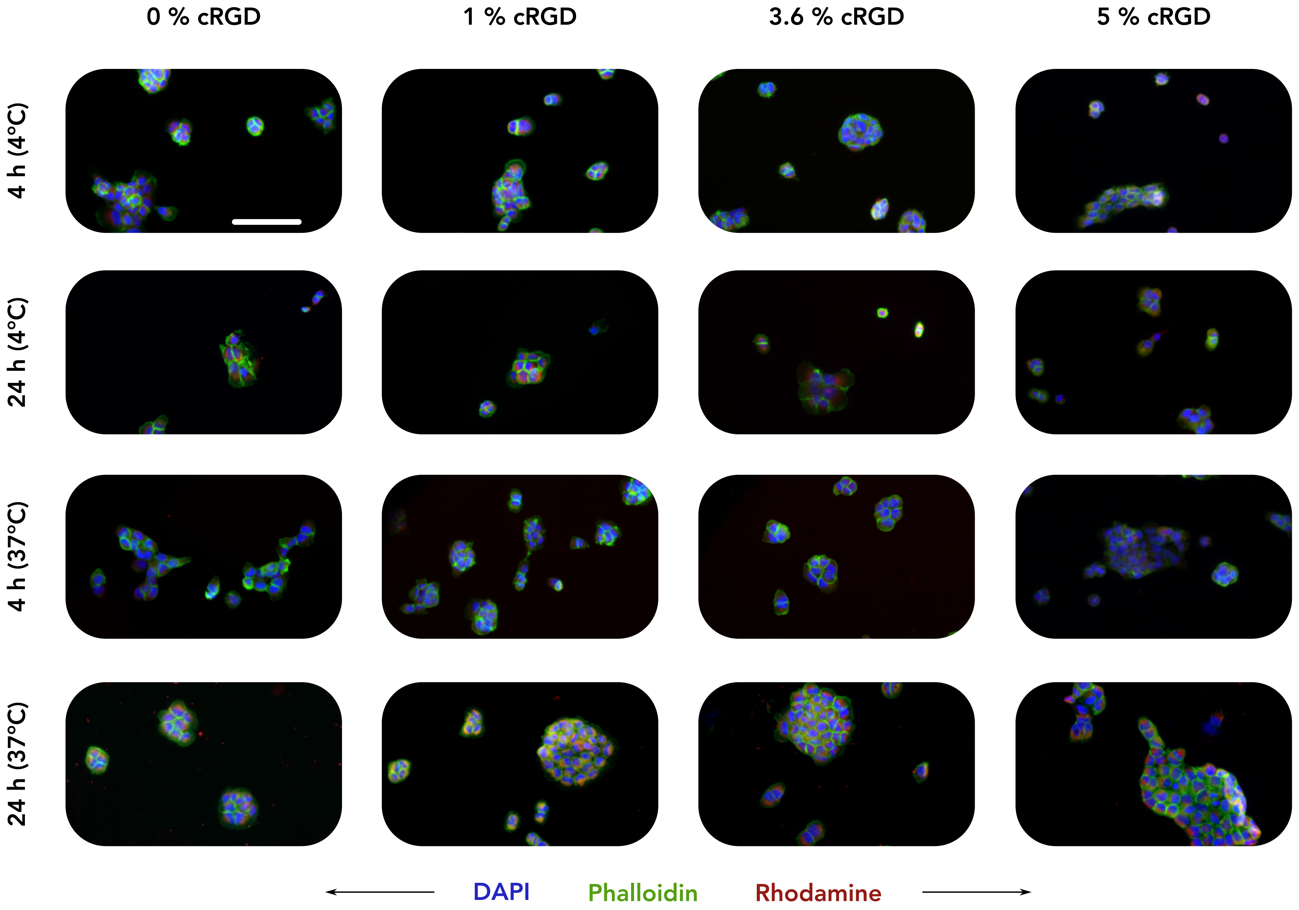


**Figure S3. Uptake of CCPM by A431 cells** (supplementary for Fig. 2, 3). A431 cells take up control and cRGD-decorated CCPM at similar extents for all incubation time-points (4, 24 h) and temperatures (4, 37^o^C). Color coding: DAPI in blue, phalloidin in green, rhodamine-labelled CCPM in red. Scale bar = 100 μm.

Supplementary Figure 4


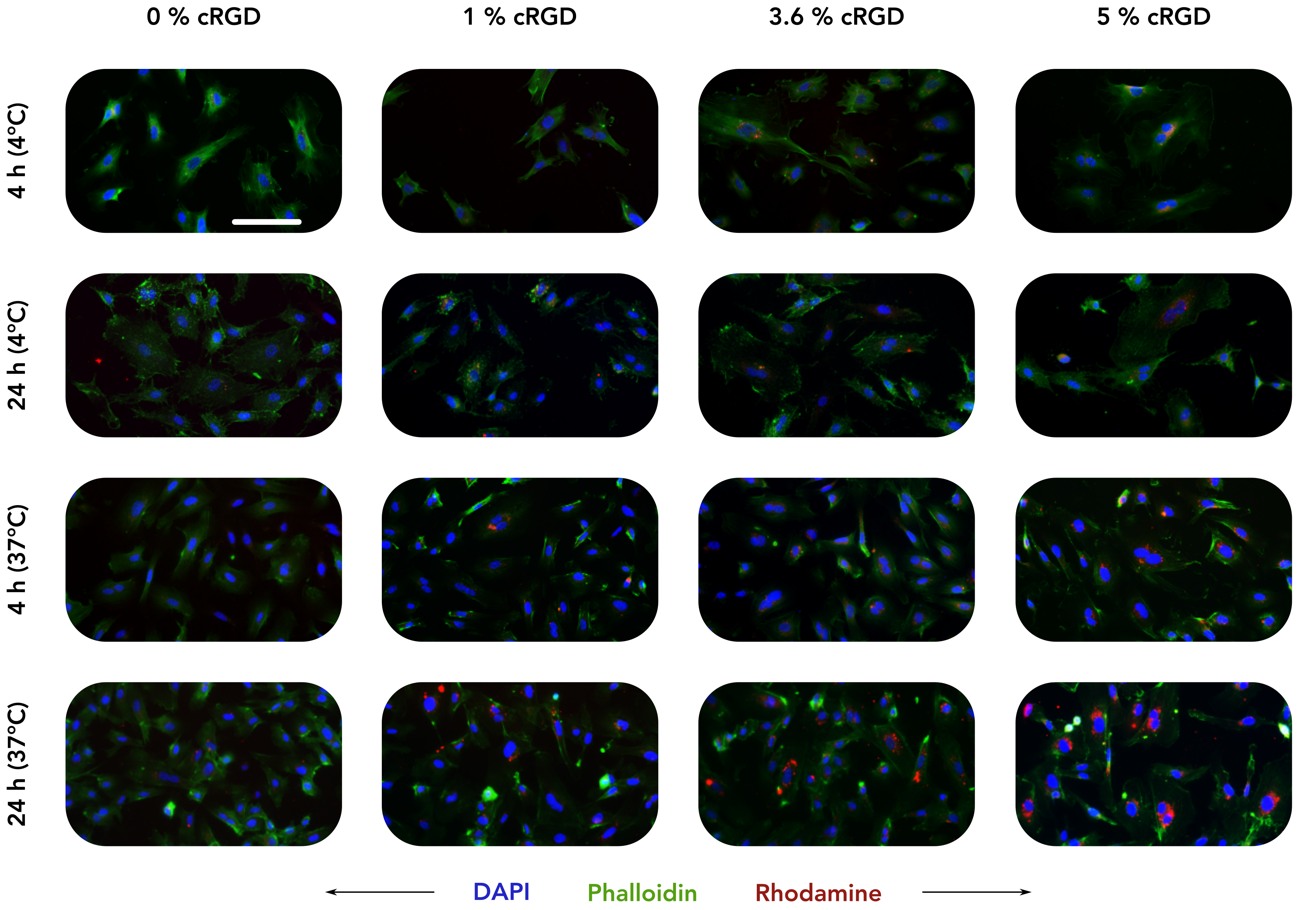


**Figure S4. Uptake of CCPM by activated HUVEC (HUVEC^+^)** (supplementary for Fig. 2, 3). HUVEC^+^ cells take up cRGD-decorated CCPM at higher extent than control CCPM. These differences are significant for higher ligand-decoration densities (3.6, 5 mol% cRGD), longer incubation time-points (24 h), and higher temperatures (37^o^C). Color coding: DAPI in blue, phalloidin in green, rhodamine-labelled CCPM in red. Scale bar = 100 μm.

Supplementary Figure 5


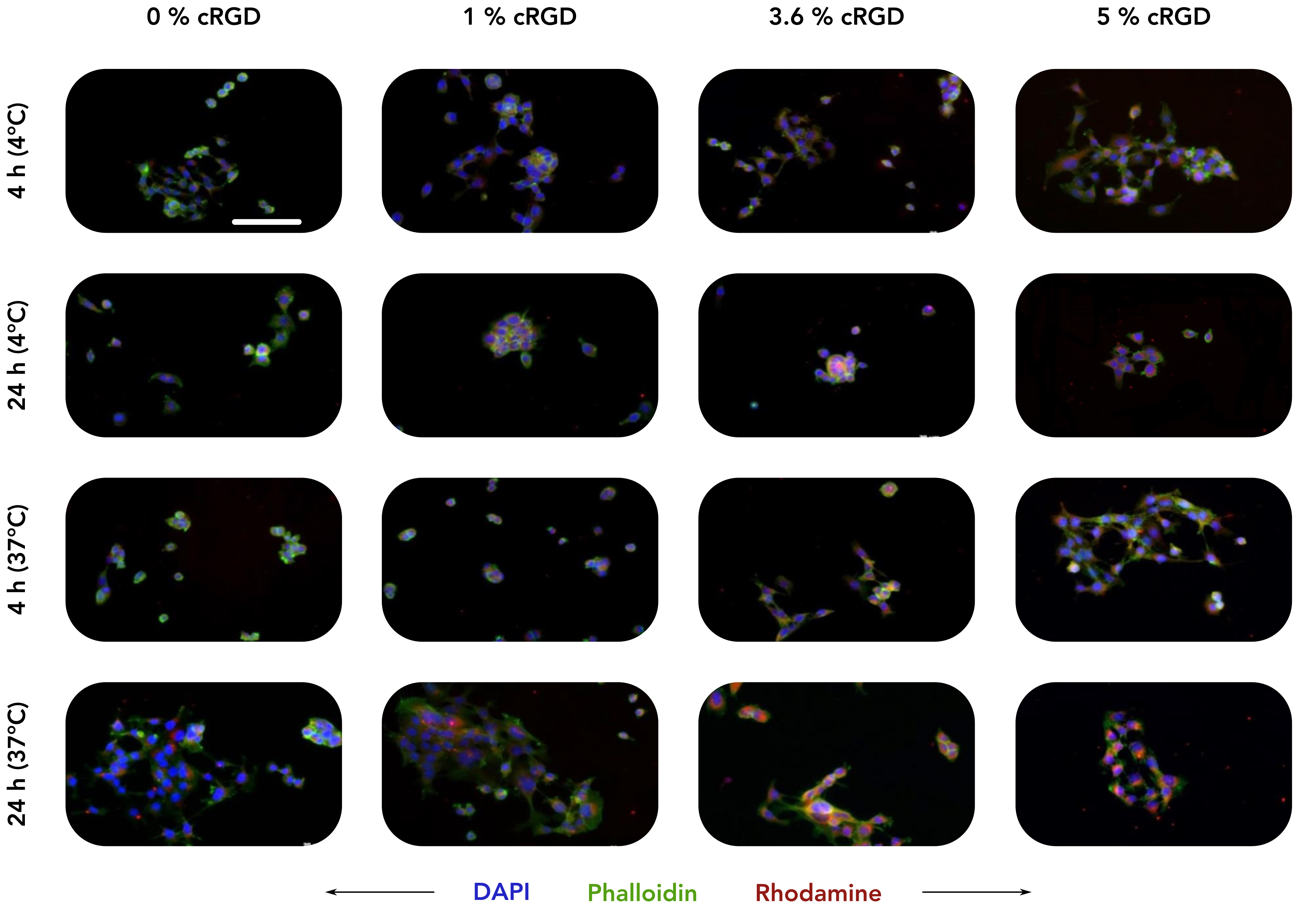


**Figure S5. Uptake of CCPM by 4T1 cells** (supplementary for Fig. 2,3). 4T1 cells take up cRGD-decorated CCPM at higher extent than control CCPM. These differences are significant for higher ligand-decoration densities (5 mol% cRGD), longer incubation time-points (24 h), and higher temperatures (37^o^C). Color coding: DAPI in blue, phalloidin in green, rhodamine-labelled CCPM in red. Scale bar = 100 μm.

Supplementary Figure 6


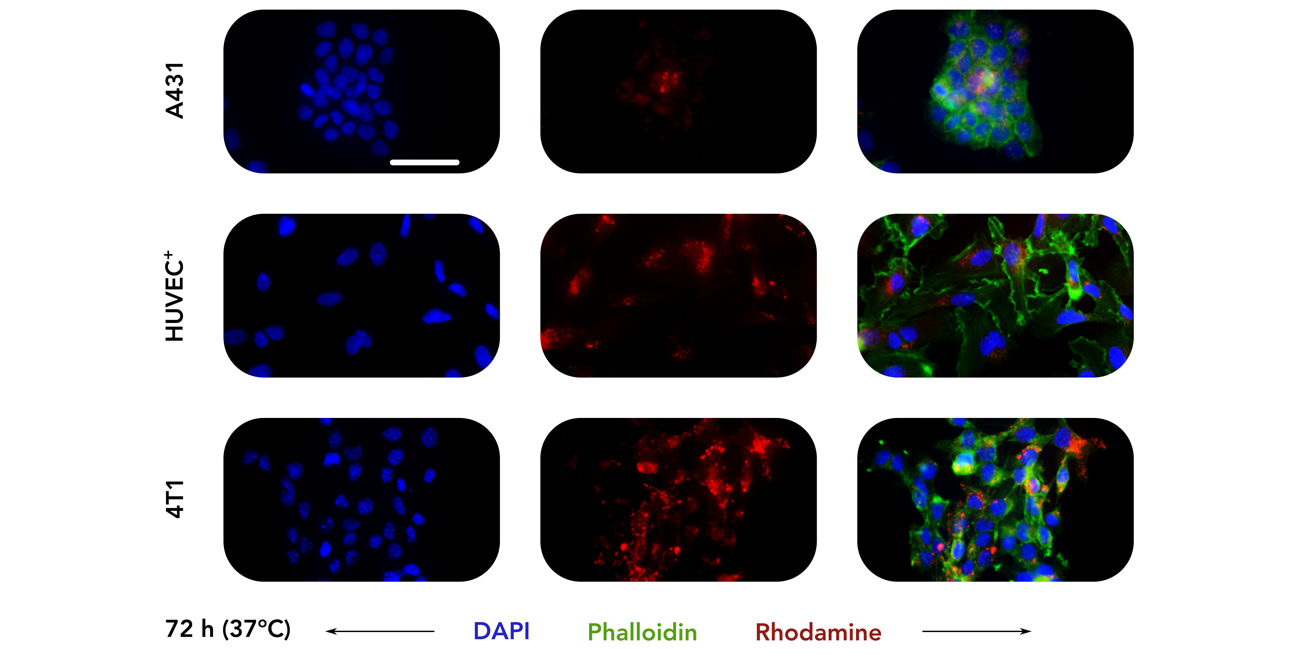


**Figure S6. Uptake of 5 mol% cRGD-decorated CCPM by 4431, activated HUVEC (HUVEC^+^), and 4T1 cells after 72 h of incubation** (supplementary for Fig. 4). 5% cRGD-CCPM are taken up at a higher extent by the cell lines expressing high levels of α_v_β_3_-integrin, i.e., HUVEC^+^ and 4T1. Color coding: DAPI in blue, phalloidin in green, rhodamine-labelled CCPM in red. Scale bar = 100 μm.

Supplementary Figure 7


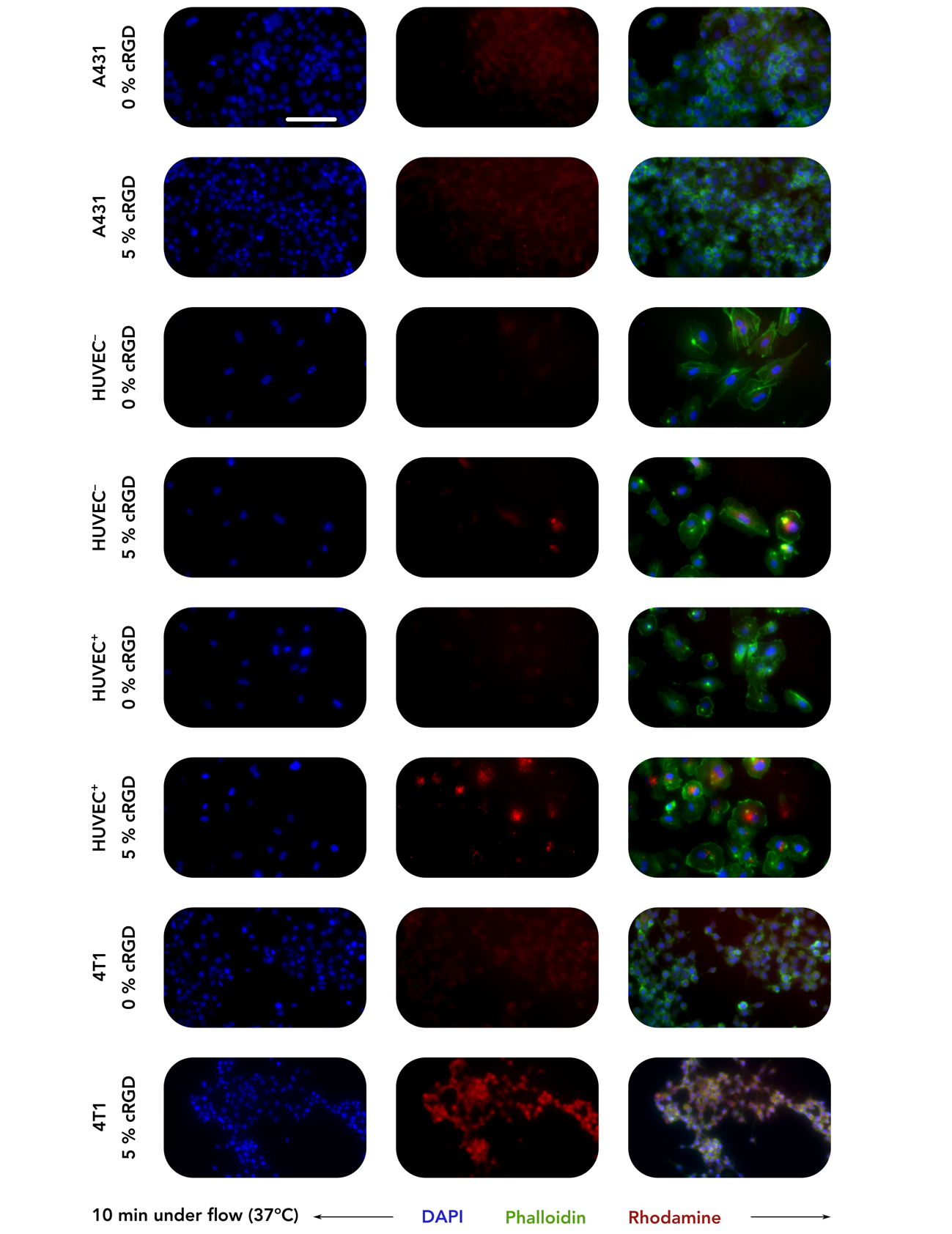


**Figure S7. Uptake of control and 5 mol% cRGD-decorated CCPM by 4431, activated HUVEC (HUVEC^+^), and 4T1 cells after 10 min incubation under flow** (supplementary for Fig. 5). 5% cRGD-CCPM are taken up at a higher extent by α_v_β_3_-integrin^high^ cells, i.e., HUVEC^+^ and 4T1, while control CCPM display a low uptake at similar extents by all four cell lines. Color coding: DAPI in blue, phalloidin in green, rhodamine-labelled CCPM in red. Scale bar = 100 μm.

Supplementary Figure 8


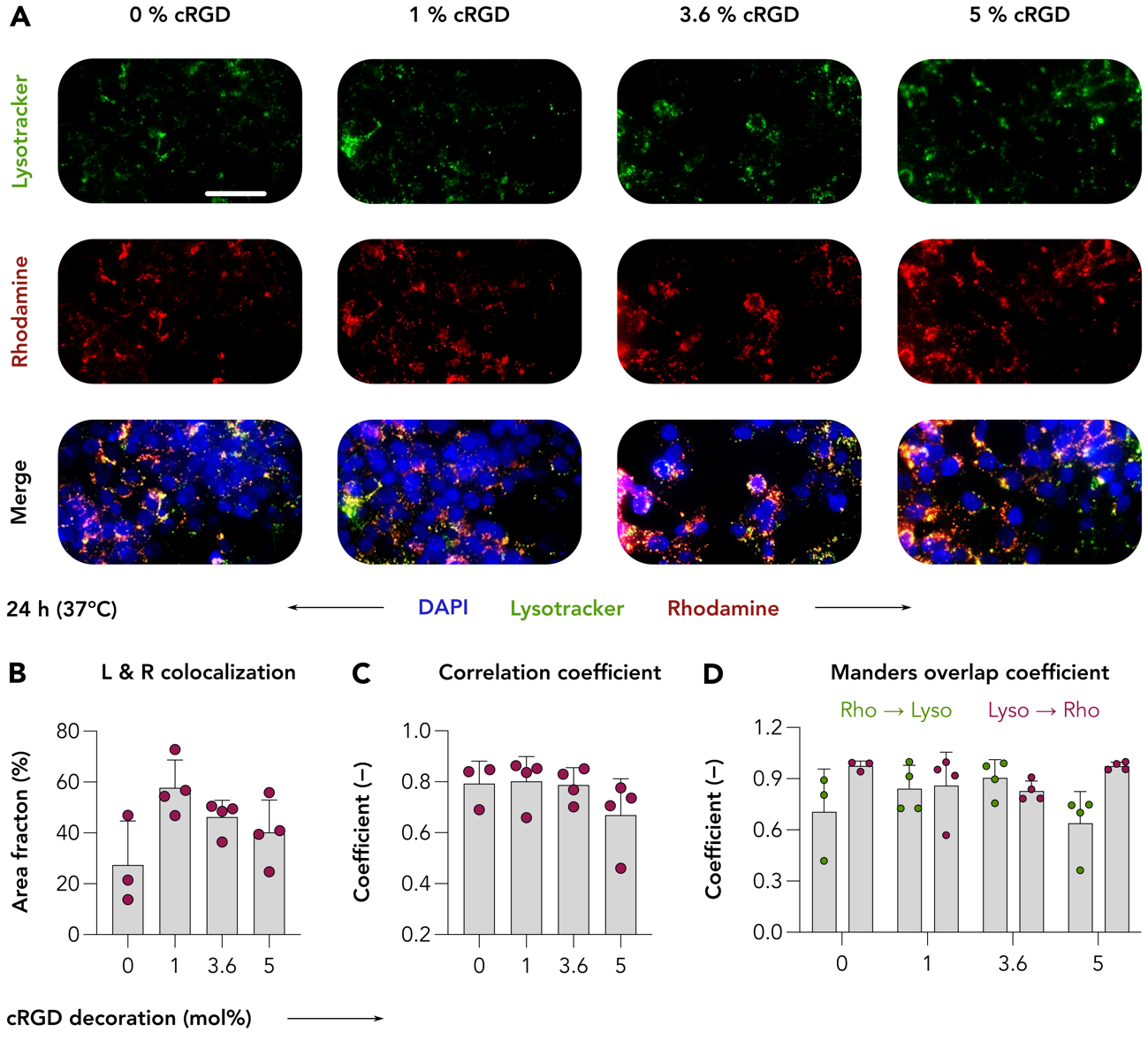


**Figure S8. CCPM internalization assessment by 4T1 cells via the LysoTracker staining** (supplementary for Fig. 6). **A.** CCPM with higher cRGD decoration densities display a higher uptake by 4T1 cells, which is in line with the higher LysoTracker signal, indicative for higher amount of lysosomes formation. Color coding: DAPI in blue, LysoTracker in green, rhodamine-labelled CCPM in red. Scale bar = 100 μm. **B-D.** Quantification of colocalization between LysoTracker and Rhodamine-CCPM, including (**B**) area fraction % of colocalization, (**C**) Pearson’s correlation coefficient, and (**D**) Manders’ overlap coefficient for the fraction of rhodamine overlapping with LysoTracker (dark green), and the fraction of LysoTracker overlapping with rhodamine (dark red).
